# Multiscale Modeling of Lytic Polysaccharide Monooxygenases in Oxidized and Reduced Redox States

**DOI:** 10.1021/acs.jpcb.6c04364

**Published:** 2026-10-15

**Authors:** Katarzyna Szleper-Shendy, Andrés M. Escorcia, Ravi Kumar, Artur Góra, Matthias Stein

**Affiliations:** † Tunneling Group, Biotechnology Centre, 49569 Silesian University of Technology, Gliwice 44-100, Poland; ‡ Molecular Simulations and Design Group, 28307 Max Planck Institute for Dynamics of Complex Technical Systems, Magdeburg 39106, Germany

## Abstract

Lytic polysaccharide monooxygenases (LPMOs) are copper-dependent oxidative enzymes whose catalytic activity depends on redox cycling of a single copper ion in the active site, coordinated by the conserved histidine brace motif. Although the static coordination geometry of the active site has been characterized crystallographically, the dynamic response of the histidine brace to Cu­(I)/Cu­(II) cycling and the role of second-sphere residues in modulating this response remain poorly understood at the atomistic level. Here, we address this gap using a multiscale computational approach combining molecular dynamics (MD) simulations, QM/MM optimization, and EPR parameter calculations, applied to two representative LPMOs: NcAA9C (AA9, axial tyrosine) and BaAA10A (AA10, axial phenylalanine). The structural response to the change in oxidation state is seen almost entirely in the exogenous ligands rather than in the histidine brace itself, with the oxidized form showing markedly higher and longer-lived water coordination. In BaAA10A, the equatorial water is anchored by a second-sphere glutamate with no counterpart in NcAA9C. QM/MM electrostatic perturbation analysis identifies Asp76 (NcAA9C) and Glu68 (BaAA10A) as the dominant stabilizing residues of the catalytic core, acting through water-mediated hydrogen-bond bridges that are more pronounced in the Cu­(II) state. Computed EPR parameters respond to the displacement of the copper site from a planar geometry, and the equatorial water position was shown to have the dominant effect, differentiating the two LPMOs.

## Graphical abstract

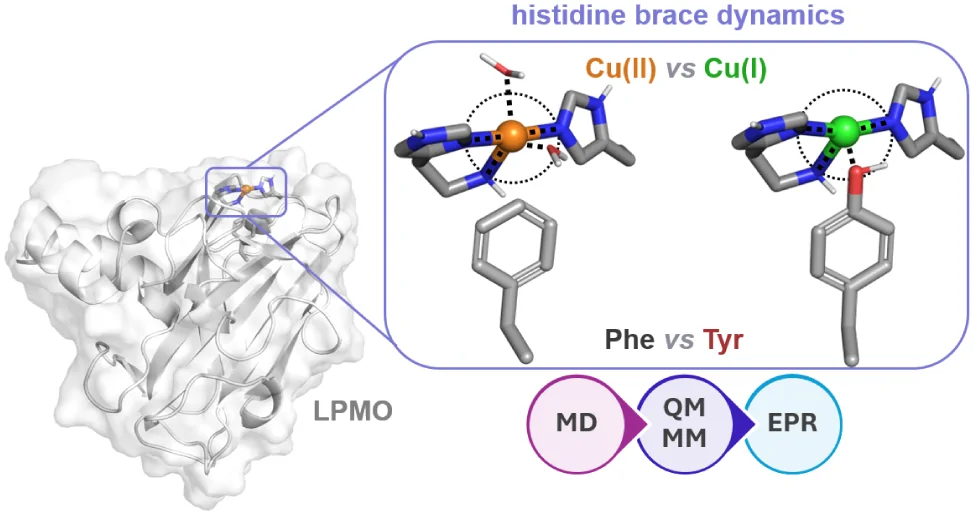

## Introduction

Lytic polysaccharide monooxygenases (LPMOs) are copper-dependent enzymes found across all domains of life.^1^ Originally identified as enzymes capable of oxidative cleavage of glycosidic bonds in recalcitrant polysaccharides,^2,3^ LPMOs disrupt polysaccharides crystallinity and strongly enhance the activity of classical glycoside hydrolases, fundamentally reshaping our understanding of enzymatic biomass conversion.^4^ Beyond their established role in biomass degradation, LPMOs have more recently been identified in diverse biological contexts, including fungal and oomycete plant pathogenesis,^5^ bacterial virulence^6,7^ and insect development,^8^ underscoring the broader biological significance of this enzyme family.

Within the Carbohydrate-Active enZYmes (CAZy) database, LPMOs are classified as auxiliary activity (AA) enzymes, which comprise redox enzymes that assist polysaccharide degradation by oxidative mechanisms, thereby enhancing the activity of classical glycoside hydrolases.^9,10^ They are currently grouped into eight families (AA9–AA11 and AA13–AA17) based on sequence and structural similarities. These families are broadly associated with species of origin and substrate specificity, with most LPMOs acting on cellulose or chitin. Despite significant diversity across families, LPMOs share a conserved catalytic architecture centered on a single copper ion.

The catalytic mechanism of LPMOs remains actively debated. Depending on reaction conditions, LPMOs have been proposed to operate either as monooxygenases using molecular oxygen or as peroxygenases utilizing hydrogen peroxide as the cosubstrate.^11^ Accumulating experimental evidence indicates that the peroxygenase pathway is significantly faster, with turnover rates several orders of magnitude higher than those observed for the O_2_-driven monooxygenase reaction, suggesting that H_2_O_2_-dependent catalysis is kinetically favored and may dominate under many physiological and *in vitro* conditions where hydrogen peroxide is available.^12,13^ Nevertheless, both pathways share a common initial step involving reduction of the resting Cu­(II) center to the catalytically competent Cu­(I) state.^14^

Central to LPMO activity is the conserved histidine brace motif, in which a single copper ion is coordinated in a distinctive, tridentate arrangement, forming a T-shaped CuN_3_ primary coordination sphere (Figure 1A). It is formed by the N-terminal histidine, which coordinates copper via both its main chain nitrogen atom and its imidazole side chain, and by another histidine residue which contributes its side chain.^15–18^ In the Cu­(II) resting state, these three nitrogen donors define the equatorial plane, often augmented by one weakly bound ligand such as a water molecule. The two axial positions above and below the equatorial plane are occupied by a water ligand and a tyrosine residue, whose phenolic oxygen is within or near coordination distance of the copper ion, or, in LPMOs featuring phenylalanine instead of tyrosine, by a single water molecule, leaving one axial position unoccupied, since phenylalanine acts as a second-sphere residue without direct metal coordination. This axial coordination further modulates LPMO structure and reactivity and differs across enzyme families.^19^ Most LPMO families (AA9, AA11, AA13, AA14, AA16, AA17 and some AA15 members) contain a tyrosine at this position, while AA10 LPMOs and most AA15 members instead feature a phenylalanine. The oxidized Cu­(II) center in LPMOs can adopt a range of geometries, including trigonal bipyramidal, distorted square-pyramidal, seesaw or elongated octahedral configurations, reflecting the flexibility of axial ligand coordination and the protein environment.^20^ Upon reduction to Cu­(I), the geometry relaxes toward a T-shaped arrangement, with characteristic changes in Cu-ligand angles and coordination distances.^18,20^ These geometric transitions underpin the redox cycling essential for LPMO catalysis and contribute to family dependent differences in substrate specificity and reactivity. Notably, these structural changes are relatively modest compared to those observed in many synthetic copper complexes, highlighting the rigidity and finely tuned nature of the LPMO active site.^21^

**Figure 1:**
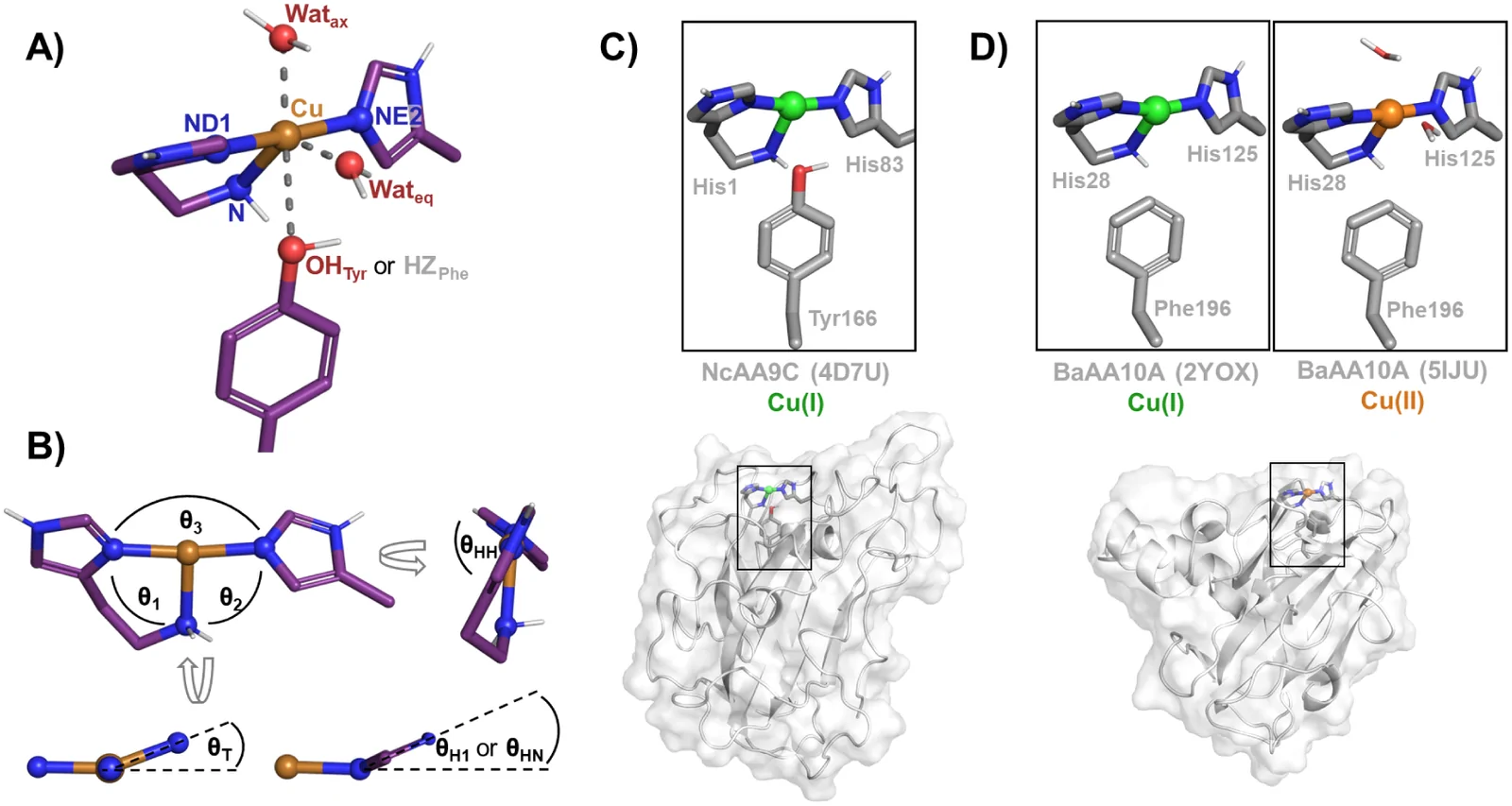
(A) LPMO histidine brace. Interactions of copper with the nitrogen atoms of the histidine brace are shown as sticks, interactions with axial and equatorial water molecules and with tyrosine or phenylalanine are shown as gray dashed lines, reflecting the bonded and nonbonded treatment of these ligands in our force field parametrization. (B) Key angular parameters (θ) describing the histidine brace and atoms participating in copper coordination. θ_1_ (N–Cu–ND1), θ_2_ (N–Cu–NE2) and θ_3_ (ND1-Cu-NE2) describe the angles between the copper-coordinating nitrogen atoms. θ_HH_ is the dihedral angle between the two histidine imidazole planes. θ_T_ is the angle between Cu–NE2 vector and the plane defined by Cu, N and ND1. θ_H1_ and θ_HN_ describe the angle between Cu–N vector and imidazole planes of both histidines. (C) NcAA9C (PDB: 4D7U, Cu­(I)) with a close-up of the histidine brace active site showing His1, His83, and Tyr166. (D) BaAA10A active site from two crystal structures with Cu­(I) (PDB: 2YOX) and Cu­(II) (PDB: 5IJU), showing His28, His125, and Phe196. Full protein views show surface and cartoon representations with a zoom in on the active site. Cu­(I) is shown in green, Cu­(II) in orange.

Structural descriptors of the histidine brace geometry, including copper-ligand distances (Figure 1A) and key angular parameters (θ, Figure 1B) defining the coordination sphere, have been systematically characterized in multiple LPMO crystal structures and summarized in the literature.^20,22,23^ X-ray photoreduction experiments on EfaAA10A,^22^ as well as LsAA9A and TaAA9A,^23^ demonstrate that gradual reduction of the active-site Cu­(II) is accompanied by systematic changes in coordination geometry, including elongation and eventual loss of solvent ligands, shifts in Cu-ligand distances and changes in θ angles describing the histidine brace. While such crystallographic data allows structural trends to be correlated with changes in copper oxidation state and provide valuable reference points for interpreting coordination geometries observed in crystal structures, they also highlight an inherent limitation of X-ray crystallography for unambiguous oxidation-state assignment in LPMOs. Owing to radiation-induced reduction and the highly conserved nature of the histidine brace coordination motif, Cu­(II) and Cu­(I) states may adopt closely related geometries^22,23^ and therefore oxidation states inferred solely from static crystallographic snapshots must be interpreted with caution.

Electron paramagnetic resonance (EPR) spectroscopy provides a powerful and complementary experimental probe of the LPMO active site.^24^ Because Cu­(I) is EPR silent whereas Cu­(II) is paramagnetic, EPR measurements directly report on the presence and electronic structure of the oxidized copper center. Extensive EPR studies of LPMOs have revealed predominantly type-2 rhombic Cu­(II) signals with anisotropic g- and A-tensor parameters, characteristic for distorted histidine-brace coordination with a sensitivity to axial ligands such as water and tyrosine (e.g., typical spectra with g_*z*_ > g_*x*_ ≈ g_*y*_ and large A_*z*_ values).^25,26^ Substrate binding, ligand exchange or modification of the axial environment can perturb these EPR parameters. Multifrequency CW-EPR shows rearrangement of the copper coordination sphere upon chitin binding in AA10 LPMOs, with associated changes in spin Hamiltonian parameters indicative of geometric and electronic reorganization of the active site.^24^ Advanced EPR techniques further indicate that protonation state and hydrogen bonding patterns near the histidine brace influence coordination environment and thereby spectral features.^27^

LPMOs have also been studied computationally using quantum mechanics (QM), quantum mechanics/molecular mechanics (QM/MM), and molecular dynamics (MD). These methods provide valuable insights into the reaction mechanism and interactions of the copper center with substrates and cosubstrates at a level which is not accessible by experiments, thus helping establish fundamental mechanistic proposals for LPMOs. By applying density functional theory (DFT) to the LPMO active site, reactive intermediates and oxygen activation pathways have been characterized. Species such as Cu-superoxo and Cu-oxyl have been identified which may participate in substrate oxidation via oxygen rebound mechanisms and hydrogen abstraction pathways.^28–30^ QM/MM studies, incorporating the surrounding protein environment, have been used to simulate key catalytic steps, such as homolytic peroxide cleavage and the formation of caged hydroxyl radicals.^31^ Classical MD simulations were applied less extensively to LPMOs than QM-based approaches, primarily due to the challenges associated with accurately describing the copper-coordinated histidine brace using force fields. MD studies have focused mainly on global protein dynamics, substrate binding modes and surface interactions with crystalline polysaccharides, providing insight into how LPMOs recognize and bind insoluble substrates. For example, MD simulations have been used to explore cellulose and chitin binding surfaces, loop flexibility and substrate positioning in AA9 and AA10 LPMOs, highlighting the importance of extended surface interactions and aromatic residues for productive binding.^32,33^ In MD simulations, the copper binding site description approaches include nonbonded copper models stabilized by distance restraints between the metal ion and coordinating residues (e.g., crystallographic waters and tyrosine),^34^ as well as bonded parametrization for metal–ligand interactions for LPMO active sites and other copper enzymes with relevant intermediates.^35,36^

Despite extensive structural and spectroscopic characterization of the LPMO active site, the dynamic response of the histidine brace to Cu­(I)/Cu­(II) redox cycling remains underexplored at the atomistic level and the role of the axial ligand further to modulate this response has not been systematically examined.

In this work, we used a combination of computational approaches to study the structural dynamics, as well as structural and electronic properties of LPMO active site across different copper oxidation states. We selected NcAA9C from *Neurospora crassa* ^37^ (Figure 1C) and BaAA10A from *Bacillus amyloliquefaciens* ^38,39^ (Figure 1D), as they are well characterized members from the two most abundant LPMO clades (AA9 and AA10), with available crystal structures without post-translational modifications and characteristic EPR signatures, providing a robust basis for computational validation. Additionally, AA9 and AA10 enzymes differ in the residue occupying the axial position (tyrosine and phenylalanine, respectively), allowing us to investigate how its character influences active site geometry and dynamics. MD simulations were performed for both LPMOs using force field parameters specifically developed and validated for the histidine brace in both Cu­(I) and Cu­(II) states, with copper–histidine interactions treated by bonded terms and variable ligands by nonbonded interactions. Representative MD snapshots selected by clustering were optimized by QM/MM, enabling an accurate assessment of copper coordination geometry and its sensitivity to oxidation state. A QM/MM electrostatic perturbation analysis was performed to identify residues contributing most to the electrostatic stabilization of the copper site. Finally, cluster models derived from QM/MM-optimized structures were used to calculate g-tensors, copper hyperfine couplings and ^14^N superhyperfine couplings and assess their sensitivity on sampled geometries.

## Methods

### Preparation of the Systems for MD Simulation

Crystal structures of LPMO from *Neurospora crassa* (NcAA9C, PDB code with Cu­(I) bound 4D7U^37^) and LPMO from *Bacillus amyloliquefaciens* (BaAA10A/BaCBM33, PDB code with Cu­(I) bound 2YOX^38^ and with Cu­(II) bound 5IJU^39^) were retrieved from Protein Data Bank. All three LPMOs are monomeric single-domain enzymes, and the asymmetric unit of each crystal structure contains two noncrystallographic symmetry-related copies of the monomer. Chain A was selected in each case, as both chains showed equivalent sequence completeness and structural quality. The copper ions and crystallographic water molecules associated with chain A were retained. For NcAA9C protein, the Cu­(I)-bound crystal structure (4D7U) was used as the input structure for independent simulations of both oxidation states. Protonation states of standard residues at pH 7.0 were assigned using PROPKA^40^ as implemented in PDB 2PQR.^41^

Input files for MD simulations of both proteins in both copper oxidation states were prepared using tleap within AmberTools25.^42^ The force field parameters for the histidine brace motif with Cu­(I) and Cu­(II) were derived through the MCPB.py workflow,^43^ which automates QM-based parametrization of metal centers in Amber software,^42,44,45^ including Hessian-derived bonded terms. The correct formal charge of +1 and +2 is preserved for the histidine brace complex in the reduced and oxidized states, respectively, while allowing for oxidation-state-dependent charge redistribution across the copper ion and coordinating histidines (Supplementary Table S1). In addition, selected bond and angle parameters differ between the two oxidation states, with the Cu­(II) model exhibiting shorter equilibrium Cu–N distances and larger force constants than Cu­(I), reflecting a more rigid coordination environment (Supplementary Table S2). All remaining force field terms, including dihedral and nonbonded parameters, are identical in both models, enabling a controlled and physically motivated comparison of Cu­(I) and Cu­(II) dynamics without the need for distance restraints (see Supporting Information for the details of the used parametrization).

For all the remaining residues, the ff19SB force field^46^ was applied. While solvating the system, positions of crystallographic water molecules were retained. Each system was solvated in the OPC four-point water model^47^ within a truncated octahedral box under periodic boundary conditions, extending at least 10 Å from any solute atom. Systems were subsequently neutralized by addition of Na^+^ or Cl^–^ counterions as required, no additional salt was included.

### MD Protocol

All MD simulations were performed using Amber24.^42,44,45^ Minimization and equilibration of all four systems were performed following the protocol of Roe and Brooks.^48^ The protocol consisted of multiple minimization and equilibration stages with progressively reduced positional restraints on heavy atoms. Energy minimization was first performed using the steepest descent algorithm with strong harmonic restraints on all heavy atoms (5 kcal mol^–1^ Å^–2^), followed by additional minimization rounds with restraints stepwise reduced to 2, 0.1, and finally 0 kcal mol^–1^ Å^–2^. The systems were then gradually heated from 0 to 300 K over 15 ps in the NVT ensemble with initial velocities assigned from a Maxwell–Boltzmann distribution at 300 K. This was followed by a series of short NPT equilibration stages with backbone and heavy-atom restraints progressively released. A final unrestrained NPT equilibration of 10 ns was performed to ensure convergence of thermodynamic parameters and stability of the protein conformation.

Following equilibration, three independent 100 ns production runs were performed for each system in the NPT ensemble. Each replica was initiated from the final equilibrated coordinates with a new set of initial velocities independently drawn from a Maxwell–Boltzmann distribution at 300 K. A 2 fs time step was used. The temperature was maintained at 300 K using the Langevin thermostat^49^ with a collision frequency of 5 ps^–1^, and the pressure regulated at 1 bar via a Monte Carlo barostat^50^ with a pressure-coupling interval of 100 steps. Long-range electrostatics were treated with the particle-mesh Ewald method, with a real-space cutoff of 9 Å applied to both electrostatic and van der Waals interactions. Bonds involving hydrogen atoms were constrained using the SHAKE algorithm.^51^ Trajectory frames were saved every 10 ps. Simulations were run with pmemd.cuda.

### Analysis of MD Trajectories

Postsimulation, the three production replicas per system were merged, centered and RMS-fitted to the first frame based on Cα atoms to produce a single processed trajectory using CPPTRAJ within AmberTools25.^42^ Global conformational changes were assessed by calculating the root-mean-square fluctuation (RMSF) of backbone atoms (N, Cα, C, O) per residue over the merged trajectory. Local structural stability of the active site surrounding was assessed by calculating the root-mean-square deviation (RMSD) of backbone atoms of residues within 7 Å of the active site, fitting all frames to the first frame. The active site geometry and structural fluctuations were analyzed using MDAnalysis,^52,53^ focusing on key interatomic distances, angles and interactions with water molecules. Water occupancy at the axial and equatorial coordination positions was quantified as the fraction of trajectory frames in which a water oxygen was found within 3.5 Å of the copper center, calculated over the full production trajectories. Water exchange events were defined as changes in the identity of the nearest water molecule at each coordination position between consecutive frames, providing a measure of water ligand mobility and coordination stability. Coordination distances (Cu–N, Cu–ND1, Cu–NE2, Cu–O_wat_ of the axial and equatorial water molecules and Cu-axial residue) and angles describing the histidine brace geometry (θ_1_, θ_2_, θ_3_, θ_HH_, θ_T_, θ_H1_, θ_HN_) were extracted as descriptors of the metal site environment (Figure 1A,B). These 13 parameters were standardized to remove unit bias and subjected to k-means clustering using scikit-learn.^54^ The clustering was used to sample the accessible conformational space evenly rather than to identify discrete metastable states. Cluster-validity criteria evaluated over k = 2–20 show no well-defined optimum for any of the four systems: the silhouette coefficient is highest at k = 2–3 and falls to 0.07 at k = 10, the inertia curve shows only a weak knee at k = 6–7, and the Davies-Bouldin index is minimal at k = 12–16. The criteria therefore point to different values of k, and none indicates well-separated clusters, consistent with a continuous rather than partitioned conformational space. The number of clusters is accordingly a choice of sampling resolution, balanced against the cost of the subsequent QM/MM and EPR calculations. Ten clusters were used; a convergence analysis is given in Supplementary Figure S2, showing that the marginal improvement from additional clusters falls to the level of the clustering’s own seed-to-seed variability at k ≈ 10, and that the ten representatives match the full-trajectory descriptor distributions more closely, and far more consistently, than ten randomly selected frames. For each cluster, the trajectory frame closest to the cluster centroid in feature space was chosen for subsequent QM/MM optimization. Cluster populations and the distribution of selected frames across simulation time are shown in Supplementary Figures S3–S4. In total, for each protein and copper oxidation state, 10 MD frames were selected. For comparison, the crystal structures were also subjected to the same QM/MM optimization.

### QM/MM Calculations

QM/MM geometry optimizations were performed using ChemShell 3.7.0 ^55^ with the DL-FIND optimizer.^56^ The QM region was treated with TURBOMOLE^57^ using the PBE0 functional,^58^ def2-TZVP basis set^59,60^ and D3 dispersion corrections.^61^ An electrostatic embedding approach was used such that the QM region explicitly experiences the electrostatic potential of the surrounding MM environment. The QM charge and spin multiplicity were set according to the copper oxidation state: for Cu­(I), charge of +1 and multiplicity equal to 1 (singlet); for Cu­(II), charge of +2 and multiplicity equal to 2 (doublet). The MM region was described with DL_POLY^62^ using the ff19SB force field. The QM/MM boundary was treated using hydrogen link atoms with a charge-shifting scheme^63^ to avoid overpolarization of the QM region at the boundary.

The QM region of the NcAA9C system with Cu­(I) comprised the copper ion, His1, backbone atoms of Thr2, as well as the side chains of His83 and Tyr166 residues. For BaAA10A system with Cu­(I), the QM region consists of copper ion, His28, backbone atoms of Gly29 and the side chains of His125 and Phe196 residues. These QM regions have 49 atoms in total. For systems comprising Cu­(II), the QM region was expanded to include the two water molecules closest to the copper ion, giving 55 atoms. Moreover, an active region was defined as comprising all residues and water molecules containing at least one atom within 6 Å of the QM region, while ensuring consistency as to the total number of atoms in this region across different MD snapshots. Water molecules beyond 10 Å of the QM region were deleted. The positions of all atoms outside the active region were kept fixed during the optimizations.

To get a quantitative measure of the influence of residues around the active site on the QM region, we performed electrostatic perturbation analysis. In this approach, the point charges of residues in the MM region are set to zero and a QM/MM single point energy calculation is performed. This generates a perturbed QM energy, such that the electrostatic contribution of the residue in the MM region to the stabilization of the QM region can be calculated as the difference between the original QM energy and the perturbed QM energy (eq 1). Residues within 7 Å of the QM region were selected for this purpose. In addition, the total energy contribution of the MM region to the electrostatic stabilization of the QM region was calculated by performing gas-phase single point energy calculations of the QM region geometry and subtracting it from the original QM energy (eq 2). A negative ΔE indicates that the residue stabilizes the QM region, while a positive value suggests a destabilizing effect.

1 $$\Delta E_{\mathrm{residue}}=E_{\mathrm{SP}}^{\left(\mathrm{electrostatic}\right)}-E_{\mathrm{SP}}^{\left(\mathrm{electrostatic}+\mathrm{residue charges}=0\right)}$$

2 $$\Delta E_{\mathrm{total}}=E_{\mathrm{SP}}^{\left(\mathrm{electrostatic}\right)}-E_{\mathrm{SP}}^{\left(\mathrm{gas‐phase}\right)}$$

### Calculations of EPR Parameters

The QM regions from the QM/MM optimizations of Cu­(II)-bound LPMOs were used for subsequent EPR parameter calculations. Unsaturated bonds in the QM region were capped with hydrogen atoms. Only the hydrogen atoms were reoptimized using ORCA 6.0.1,^64^ employing the same level of theory as in the QM/MM step (PBE0 functional with the def2-TZVP basis set and Grimme’s D3 dispersion correction). Only the Cu­(II) systems were considered, as Cu­(I) is EPR-silent.

For the g-tensor calculations, we adopted the PBE0-DH double-hybrid density functional,^65^ which has been shown to be among the most accurate functionals for predicting g-tensors of Cu­(II) complexes, as established in a recent comprehensive benchmark study.^66^ The recontracted ZORA-def2-TZVPP basis set^67^ was used for copper and ZORA-def2-TZVP was used for all other atoms, together with their corresponding fully decontracted def2/J auxiliary basis sets.^59^ Scalar relativistic effects were described using the zeroth-order regular approximation (ZORA).^68,69^ Copper and nitrogen hyperfine couplings were obtained with B3PW91 and an aug-cc-pVTZ-J basis on copper^70,71^ with def2-TZVP on all other atoms, with all orbital basis sets fully decontracted and without a scalar relativistic Hamiltonian, following the protocol established for Cu­(II) hyperfine couplings.^72^ The resolution-of-identity (RI) approximation^73^ and the chain-of-spheres approximation to exact exchange (COSX)^74^ were employed in all calculations to enhance computational efficiency. All calculations used tight SCF convergence criteria and the DefGrid3 integration grid. Spin–orbit coupling effects were described using the spin–orbit mean-field (SOMF) operator^75^ within the 1X approximation^76,77^ (implemented as SOCType 3 and SOMFType 1X in ORCA), including picture-change corrections where a relativistic Hamiltonian was used. The SOC integrals were evaluated using the RI approximation for the Coulomb contribution and one-center exact exchange for the exchange contribution, employing SOCFlags 1, 3, 3, 0, in accordance with established protocols for accurate EPR g-tensor calculations.^66^ A_*z*_ is reported as the hyperfine component projected onto the g_*z*_ principal axis, A_*z*_ = ê_gz_·A·ê_gz_, which is the quantity corresponding to the splitting measured in the parallel region of the spectrum. The isotropic coupling is negative for a planar-type Cu­(II) site, since the singly occupied d­(x^2^ – y^2^) orbital has a node at the copper nucleus and the Fermi contact term arises entirely from its polarization of the filled inner s shells. A_iso_ is the sum of that contact term and the isotropic part of the second-order spin–orbit contribution, and the latter is almost constant across the structures (+133 ± 5 MHz), whereas the contact term weakens steadily from −232 MHz as the copper leaves the equatorial plane. For the structures retaining a negative A_iso_ this differs from the largest principal value of A by less than 1%, so the values are directly comparable with benchmark data quoted for that component, whereas for the more distorted structures, where the tensors are not coaxial, the two differ by some 45%.

For the histidine-brace nitrogens the perpendicular component A_⊥_ = (A_*x*_ + A_*y*_)/2 is reported for the same frame, since the ^14^N superhyperfine is resolved on the high-field feature rather than in the parallel region where the copper lines dominate. Because Borisova et al.^37^ reports a single unresolved splitting constant for the nitrogen donors, the mean of the three calculated nitrogen hyperfine couplings is calculated and compared with experiment.

The dependence of the calculated parameters on the coordination geometry was analyzed using two descriptors, both evaluated over the four shortest copper-ligand contacts (the three histidine-brace nitrogens and the nearest exogenous ligand). The tetrahedral distortion parameter τ_4_ = [360° – (α + β)]/141°, where α and β are the two largest donor-Cu-donor angles, takes the value 0 for a square-planar and 1 for a regular tetrahedral arrangement.^78^ The out-of-plane displacement d_Cu_ is the perpendicular distance of the copper ion from the least-squares plane defined by those same four donor atoms.

To quantify the contribution of individual ligands, single-point calculations were performed on perturbed clusters built from one representative snapshot of each enzyme, in which the equatorial water, the axial water, or both were deleted, or the displacement of Tyr166 along the Cu–O vector (for NcAA9C only). There was no reoptimization, so that the change in each parameter is attributable to the single coordinate that was altered. To test the influence of the protein electrostatics on EPR parameters, every structure was recomputed with the QM cluster embedded in the point-charge field of the complete solvated protein (3546 charges for NcAA9C, 3052 for BaAA10A).

### Multiple Structural Alignment

To assess the conservation of the second-sphere residues identified in this study, multiple structural alignments were performed separately for structurally characterized AA9 and AA10 LPMOs. Structural alignments were generated using the mTM-align tool^79^ and visualized with ESPript 3.2.^80^ Twenty-two structurally characterized LPMOs were aligned for the AA9 family and 26 for the AA10 family. Conservation at each position is reported as the fraction of aligned sequences carrying the same residue.

## Results and Discussion

### Structural Dynamics of LPMOs during MD Simulations

LPMOs remained overall stable throughout the MD simulations, except for certain long loop regions that exhibit enhanced flexibility. In NcAA9C, the L3 loop (Gly63-Pro86, 24 residues) and the extended LC loop (Gly176-Cys227, 52 residues) at the C-terminal region were particularly mobile, with RMSF values reaching up to ∼4 Å (Figure 2). In contrast, the L2 region in BaAA10A (Pro34-Asp80, 47 residues), while also relatively long, is formed by multiple helix-turn-helix motifs, which confer structural rigidity and result in an overall more stable protein, with RMSF values below 1.5 Å (Figure 2). By comparison, BaAA10A (178 residues), which has a shorter overall sequence than NcAA9C (227 residues), displayed reduced flexibility relative to NcAA9C. No significant differences in regions of residual flexibility or overall structural stability were observed between the Cu­(I) and Cu­(II) oxidation states (Figure 2). The RMSD of residues within 7 Å of the copper center remained below 1 Å throughout the simulations for both proteins, confirming that the active-site geometry is well preserved regardless of the overall differences in protein flexibility. A transient conformational change to RMSD of ∼1.5 Å was observed in one replicate of the NcAA9C Cu­(II) simulation. This was shown to originate from a backbone motion of Asp76, a second-sphere residue located on a flexible surface loop, whose backbone RMSD rises transiently from 0.6 Å to ∼4 Å. However, this does not perturb the first coordination sphere, as Cu-ligand distances and N–Cu–N angles remain unchanged to within 0.1–0.3 standard deviations of their trajectory-wide distributions. We note that a single such event across three replicas does not establish how often it occurs, as 3 × 100 ns are sufficient to show that the histidine brace coordination geometry is reproducible between replicas, but populations of slow loop motions of this kind cannot be sampled sufficiently.

**Figure 2:**
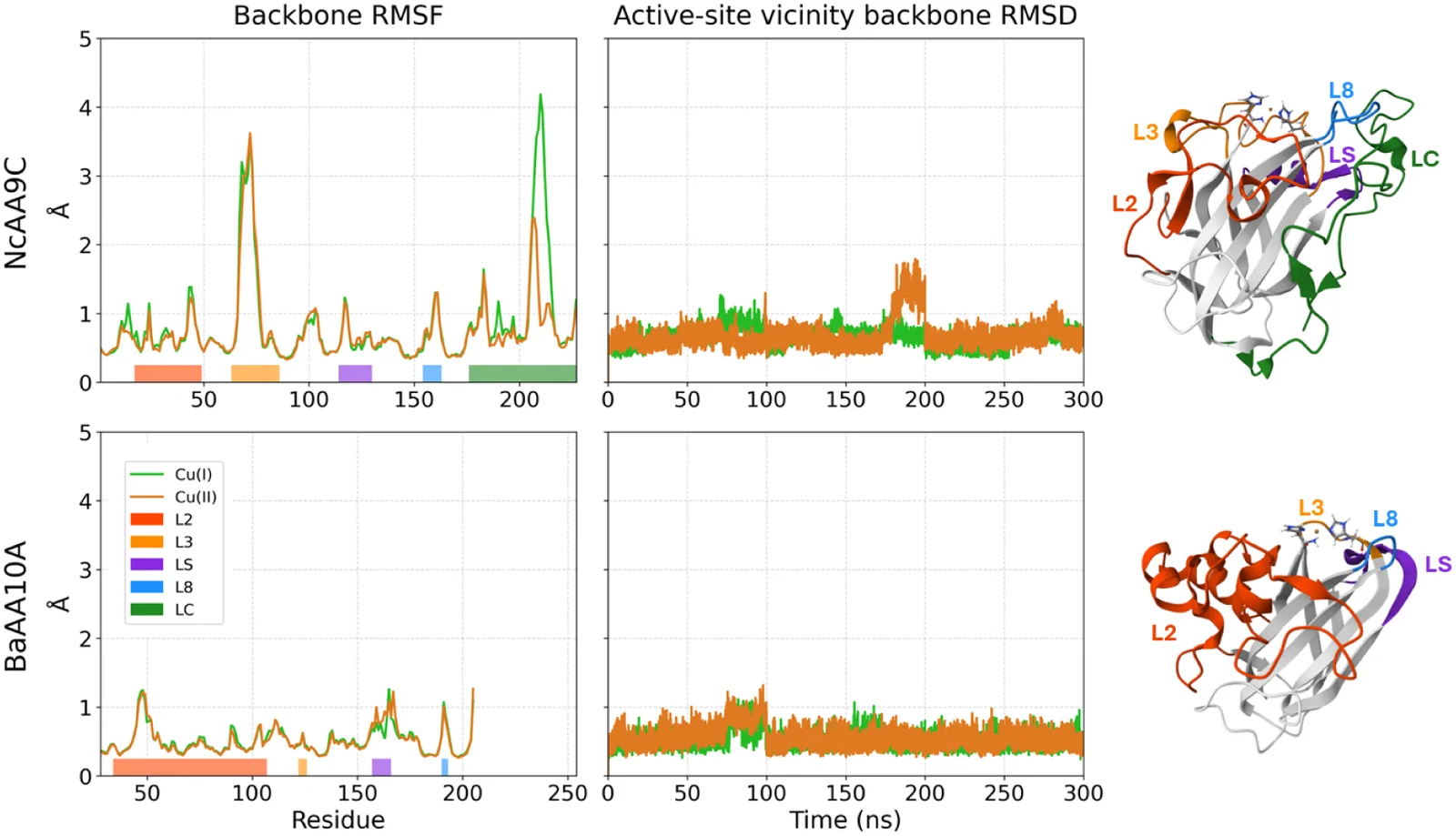
Per-residue backbone RMSF (left), backbone RMSD of active-site surrounding residues within 7 Å of the copper center (middle), and representative structures with loop regions highlighted (right) are shown for NcAA9C (top row) and BaAA10A (bottom row). In the RMSF and RMSD plots, Cu­(I) and Cu­(II) simulations are shown in green and orange, respectively. Data represents 3 merged trajectories of 100 ns replicates.

The histidine brace is described by bonded parameters, so the Cu–N distances and θ_1–3_ angles are held close to their equilibrium values by construction. The MD averages for both oxidation states and both proteins lie within 0.1 Å of the equilibrium distances and within 7° of the equilibrium angles throughout (Supplementary Table S2). Those equilibrium values match the corresponding distances and angles in the crystal structures or show only small deviations from these. The largest deviation in bond distance is approximately 0.2 Å, while that for bond angle is approximately 12° (Tables 1 and 2). The parameters reproduce the ordering of the three donors, with the two imidazole nitrogens closer to the copper than the N-terminal amine, as seen in crystallography. The Cu–N­(amine) distance shortens upon oxidation by 0.1 Å, as in the crystal structures, while Cu–N­(imidazole) distances remain almost unchanged. The response of the angles to oxidation is small; θ_2_ decreases and θ_3_ increases, as observed experimentally. These geometric shifts, while modest in absolute terms, are consistent with the role of the histidine brace in fine-tuning the Cu­(I)/Cu­(II) redox potential and supporting the redox cycling that underpins LPMO catalysis.

**Table 1:** Bond Distances (Å) of LPMOs in MD Simulations and Crystal Structures^a^

Enzyme	Cu oxidation state	Cu-His1:N	Cu-His1:ND1	Cu-His83:NE2	Cu-Tyr166:OH	Cu-Wat_ax_:O	Cu-Wat_eq_ :O
NcAA9C	Cu(I)	2.2 ± 0.1 (2.3)	1.9 ± 0.1 (2.0)	1.9 ± 0.1 (2.0)	2.8 ± 0.3 (3.0)	3.7 ± 0.4	3.6 ± 0.4
	Cu(II)	2.1 ± 0.1	1.9 ± 0.0	1.9 ± 0.0	2.7 ± 0.3	3.1 ± 0.5	3.2 ± 0.4

Enzyme	Cu oxidation state	Cu-His28:N	Cu-His28:ND1	Cu-His125:NE2	Cu-Phe196:HZ	Cu-Wat_ax_:O	Cu-Wat_eq_ :O
BaAA10A	Cu(I)	2.2 ± 0.1 (2.3)	1.9 ± 0.1 (2.0)	1.9 ± 0.1 (2.0)	2.8 ± 0.4 (2.9)	3.9 ± 0.5	3.7 ± 0.5
	Cu(II)	2.1 ± 0.1 (2.2)	1.9 ± 0.0 (2.0)	1.9 ± 0.0 (2.1)	2.9 ± 0.3 (3.0)	3.2 ± 0.5 (2.2)	2.7 ± 0.3 (2.0)

a Data from the MD simulations are reported as average ± standard deviation, while data from crystal structures are presented in parentheses.

**Table 2:** Histidine Brace Angular Parameters (θ_1_, θ_2_, θ_3_, θ_HH_, θ_T_, θ_H1_, θ_HN_) Observed for LPMOs in MD Simulations and Crystal Structures^a^

Enzyme	Cu oxidation state	θ_1_	θ_2_	θ_3_	θ_HH_	θ_T_	θ_H1_	θ_HN_
NcAA9C	Cu(I)	94.0 ± 3.4 (95.0)	104.7 ± 5.2 (99.5)	155.9 ± 4.7 (165.2)	82.5 ± 17.1 (65.4)	5.1 ± 13.6 (−2.7)	–11.0 ± 12.5 (−10.8)	–18.4 ± 8.9 (−14.5)
	Cu(II)	95.4 ± 2.4	99.5 ± 4.1	160.0 ± 4.0	85.9 ± 15.2	3.9 ± 12.5	-3.2 ± 13.4	–16.2 ± 8.5
BaAA10A	Cu(I)	95.2 ± 3.6 (97.7)	102.9 ± 5.2 (99.3)	155.7 ± 4.8 (162.6)	64.7 ± 16.4 (76.1)	5.7 ± 14.6 (3.7)	–16.1 ± 10.4 (−11.4)	–8.7 ± 11.8 (−17.6)
	Cu(II)	96.0 ± 2.4 (92.6)	97.6 ± 3.9 (95.6)	159.3 ± 4.2 (171.4)	60.9 ± 16.1 (78.5)	10.4 ± 11.5 (−2.4)	–12.5 ± 10.3 (2.3)	2.9 ± 11.4 (−14.5)

a Data from the MD simulations are reported as average ± standard deviation, while data from crystal structures are presented in parentheses.

The remaining angular descriptors are not parametrized and are therefore free to respond to the protein environment motion at finite temperature. θ_HH_, the angle between the imidazole ring planes, shows the largest variability (standard deviation 15–17°, Table 2) and the clearest difference between the two enzymes. Starting from crystallographic values of 65° in NcAA9C and 76–79° in BaAA10A, in the simulations a mean of ∼84° in NcAA9C and ∼64° in BaAA10A is seen. In each case, the crystallographic value lies within approximately one standard deviation of the simulated distribution. θ_T_, θ_H1_ and θ_HN_ likewise display substantial variation, being sensitive to distortions within the coordination sphere and to fluctuations of the surrounding residues. Full statistics for all measured distances and angles are reported in Supplementary Figure S1 and Supplementary Table S3.

The aqua ligands are described by the standard OPC water model,^47^ with no term fitted to any copper-ligand geometry. The classical description does not maintain the strict coordination at the 2.0–2.2 Å distances observed crystallographically and the average Cu–O_wat_ distance remains somewhat longer. However, the redox response is pronounced and the median axial and equatorial water distances are shorter in the Cu­(II) state by 0.5 and 0.4 Å in NcAA9C, and by 0.7 and 1.1 Å in BaAA10A, compared to Cu­(I). This reduction reflects the stronger electrostatic attraction and tighter coordination environment of the oxidized copper state. The copper charge does differ between the two models (Supplementary Table S1), but no bonded term prescribes the Cu–O distance. The most apparent difference between the enzymes can be seen for the equatorial position: in the Cu­(II) state the equatorial water sits at 2.7 Å from the copper in BaAA10A against 3.2 Å in NcAA9C, while the axial water distances are indistinguishable. The axial amino acid residues were also treated without additional parameters. In the Cu­(II) state, the copper is at 2.7 Å from the tyrosine hydroxyl oxygen in NcAA9C, whereas in BaAA10A the closest approach of the phenylalanine is at Cu–H distance of 2.9 Å, since that residue offers no polar group capable of coordinating copper. Neither distance changes appreciably with oxidation state, in contrast to the aqua ligands.

To further investigate copper solvent interactions, we analyzed the occupancy and mobility of axial and equatorial water molecules. With a 3.5 Å cutoff radius to define the first coordination shell (Supplementary Table S4), the Cu­(II) state exhibits a substantially higher water occupancy than Cu­(I). In NcAA9C, axial and equatorial site occupancies are 74% and 75% in Cu­(II) versus 36% and 41% in Cu­(I); in BaAA10A they are 74% and 97% in Cu­(II) versus 24% and 35% in Cu­(I). This consistent trend reflects tighter binding of water ligands in the oxidized state, with the most notable difference at the equatorial site in BaAA10A, where occupancy drops from 97% in Cu­(II) to only 35% in Cu­(I). The high equatorial occupancy in BaAA10A has a structural origin in the second coordination sphere. Throughout the Cu­(II) simulations the equatorial water is hydrogen-bonded to Glu68 in 77% of frames, while it is coordinated to the copper (Cu–O < 2.6 Å) in only 61%; the carboxylate therefore retains the water at the equatorial position even when it is not tightly bound to the metal. On reduction, the copper coordination is lost and the hydrogen bond substantially weakened. The axial water occupancies, by contrast, are essentially identical in the two enzymes (74% in both), so the difference between them is confined to the equatorial site rather than arising from the nature of the axial residue.

Water exchanges provide a complementary view of ligand dynamics. In all systems, axial and equatorial water molecules exchange repeatedly throughout the trajectories, highlighting the utility of the nonbonded description of solvent ligands. Exchange events are markedly more frequent in the Cu­(I) state than in Cu­(II) (Supplementary Table S5), consistent with weaker copper-water interactions and longer average Cu–O distances (see Table 1). Interestingly, BaAA10A shows exceptionally low exchange for the equatorial water in the Cu­(II) state (1,225 exchange events across all replicates, Supplementary Table S5), indicating a long-living coordination environment, consistent with the persistent hydrogen bond to Glu68 described above, whereas NcAA9C retains more dynamic hydration shells even in the oxidized state (∼16,000 events). Overall, these results demonstrate that copper oxidation state and local protein environment jointly control water ligand occupancy and mobility at the histidine brace.

These trends are well supported by structural studies. X-ray photoreduction experiments on LPMOs show that the oxidized Cu­(II) center adopts a trigonal bipyramidal geometry with two coordinated water ligands at measurable Cu–O distances, whereas reduction to Cu­(I) is associated with loss or substantial weakening of water coordination and a T-shaped geometry with distant or absent water ligands.^22,23^ Overall, the simulations show that the redox-dependent structural response of the copper site is reflected by the ligand interactions described without bonded terms. Water occupancy roughly doubles on oxidation, the equatorial Cu–O distance contracts by about 1 Å, and equatorial water exchange in BaAA10A falls by more than an order of magnitude.

The results provide confidence in the parameter set derived from QM optimizations of the active site, and hence in the reliability of other dynamic observables from the simulations. How far this description lies from the corresponding QM/MM minima is examined in the following section, QM/MM calculations.

### QM/MM Calculations

Representative MD snapshots of the systems (ten per system, selected as cluster representatives; see Methods and Supplementary Figures S2–S4) were reoptimized by QM/MM to include environmental polarization effects and a more accurate description of copper-ligand interactions (see section QM/MM calculations in Methods). MD structures are conformations sampled at finite temperature and not necessarily stationary points, so the structural changes on optimization (Supplementary Figure S5) measure how far each snapshot lies from the nearest local minimum of the QM/MM potential energy surface.

The copper–nitrogen distances shift by less than 0.05 Å on optimization. Since these bonds are constrained by the force field, this shows that embedding the site in the protein leaves them close to the model used for parametrization. The redox-dependent difference in Cu–N­(amine) distance is maintained throughout. The bond angles shift more substantially, moving toward the values observed crystallographically. θ_2_ decreases by 6° (NcAA9C) and 5° (BaAA10A), while θ_3_ increases by 6° and 10°. The remaining angles, θ_HH_ in particular, fluctuate by more than their mean change and show no systematic shift.

The aqua ligands described by nonbonded terms, which were not parametrized against any Cu-ligand geometry, shift by about 0.5 Å. In the Cu­(II) state equatorial water in NcAA9C and axial water in BaAA10A are positioned closer by 0.7 Å. The corresponding Cu­(I) distances are only minimally affected. Interestingly, the axial residues shift with no consistent direction by no more than 0.1 Å in both proteins and copper states.

These results refine the picture from the MD simulations. The QM treatment places the aqua ligands closer to the copper than the OPC model does, so the classical description systematically underestimates their binding in the oxidized state. The difference between the Cu­(I) and Cu­(II) water distances increases from 0.5–1.1 Å in MD simulations to 0.7–1.5 Å after QM/MM optimization. The conclusions drawn from MD simulations therefore rest on a trend that both descriptions reproduce. Moreover, QM/MM optimizations of the LPMOs crystal structures in absence of water molecules resulted in distorted geometries of the Cu center, which emphasizes the importance of the active site exposure to bulk water and water-mediated hydrogen bond interactions for the active site coordination and stability.

### Identification of Residues Stabilizing the Histidine Brace

Given the structural stability of the histidine brace itself, we examined the electrostatic influence of the surrounding protein environment on the catalytic core, identifying residues that contribute most to its stabilization across oxidation states. The energy contributions of protein residues to the electrostatic stabilization of the histidine brace were calculated for QM/MM optimized structures using an electrostatic perturbation approach. For the NcAA9C protein, the residue with the strongest stabilizing effect is Asp76, whose side chain is positioned on average within ∼8 Å of copper ion (Figure 3). In the Cu­(II) state, it forms water-mediated bridges with copper ion, the N and ND1 atoms of His1 and the NE2 atom of His83. In the Cu­(I) state, it forms water-mediated interactions with His1, primarily through the amine N atom, as well as imidazole ND1 and NE2. Asp76 is located in the flexible loop L3, whose length varies among AA9 family members. This residue is present in only 9% of aligned AA9 sequences. However, an adjacent position is also occupied by an Asp residue in a further 18% of sequences, indicating that a negatively charged residue in this vicinity is more broadly conserved than the single-position frequency suggests (Supplementary Figure S6).

**Figure 3:**
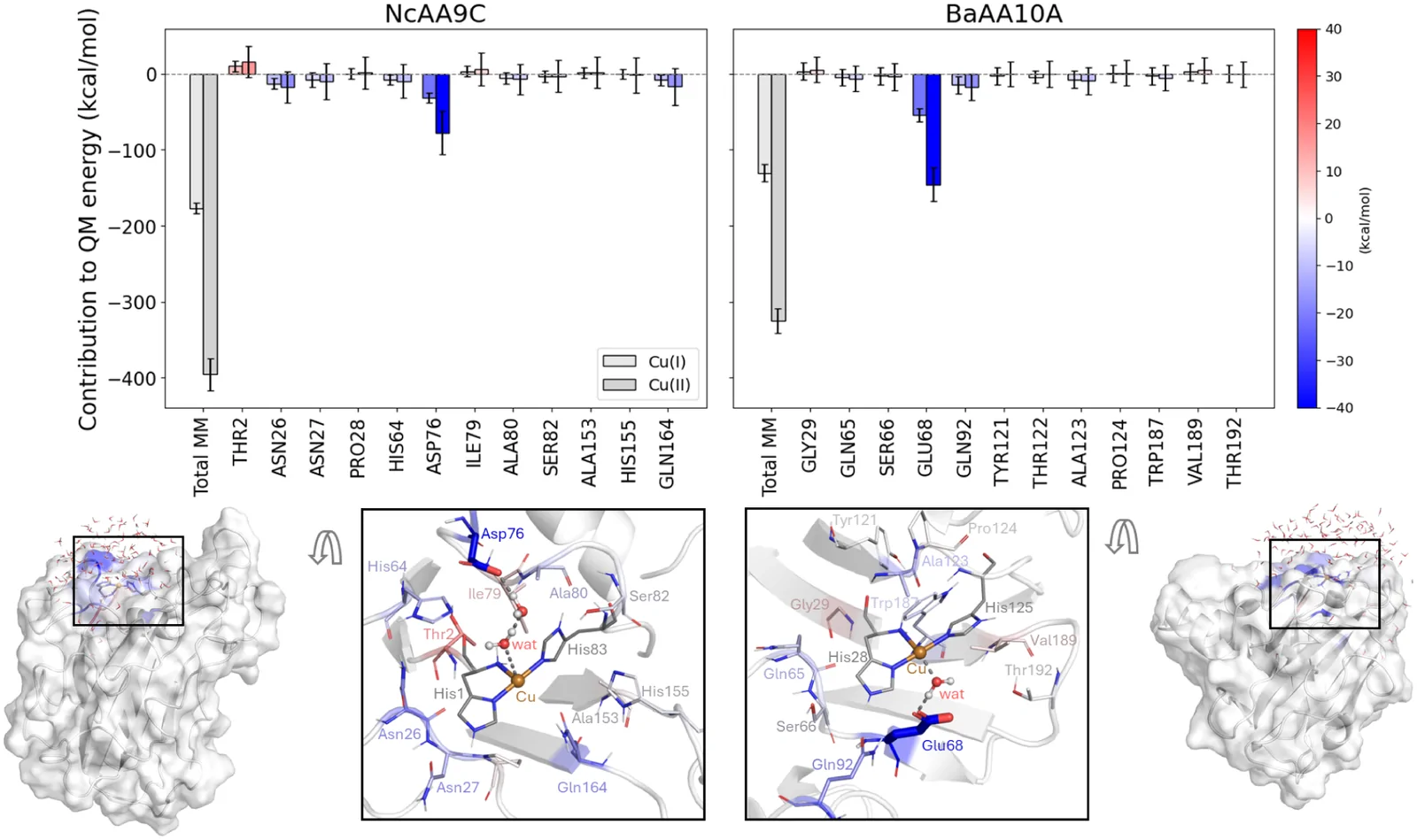
Top: Average contributions of protein residues within 7 Å of the QM region to the electrostatic stabilization or destabilization of the QM energy, calculated for QM/MM-optimized structures of NcAA9C and BaAA10A in the Cu­(I) and Cu­(II) oxidation states. The total electrostatic contribution of the MM region is also shown (Total MM). For each residue, Cu­(I) and Cu­(II) contributions are shown as adjacent bars following the blue-white-red scale according to their contribution to the QM energy (blue: stabilizing; red: destabilizing; color scale capped at ±40 kcal/mol for clarity). The total MM contribution is shown in gray. Error bars indicate standard deviations. Bottom: Active site structures of NcAA9C and BaAA10A showing residues included in the QM polarization analysis. Residues are colored on a blue-white-red scale according to their contribution to the QM energy in the Cu­(II) state. Stick thickness is proportional to the magnitude of the contribution. Copper is shown as an orange sphere, while water molecules coordinating copper are shown in CPK representation.

In the BaAA10A protein, the most stabilizing residue is Glu68, with its carboxylic side chain positioned on average within ∼4 Å of copper ion (Figure 3). Its stabilizing effect on the catalytic core is larger than that of Asp76 in NcAA9C, consistent with its closer proximity to the metal. In the Cu­(I) state, water-mediated interactions were detected with the ND1 and NE2 atoms of His28 and the NE2 atom of His125. In the Cu­(II) state, predominantly water-mediated interactions with the histidine brace were detected, including contacts with the copper ion, the ND1 and NE2 atoms of His28, and the ND1 and NE2 atoms of His125. The bridging waters include the copper-coordinated equatorial water itself, whereas those linking Asp76 to the NcAA9C active site lie outside the first coordination shell. In half of the snapshots the carboxylate additionally makes a direct van der Waals contact with the NE2 atom of His28 at 3.2 Å, without forming a hydrogen bond. The Glu68 residue in BaAA10A is situated in the structurally rigid L2 region, where it is more conserved, exhibiting 46% sequence identity across the aligned AA10 sequences (Supplementary Figure S7).

Notably, both the individual stabilizing effect of these negatively charged residues and the global stabilization by protein residues are consistently greater in the Cu­(II) state for both proteins, reflecting an enhanced stabilizing electrostatic interaction of the higher oxidation state. Noncharged residues in the vicinity of the active site do not exhibit comparably strong effects, consistent with the fact that Asp76 in NcAA9C and Glu68 in BaAA10A represent the closest charged residues to the copper center and therefore exert the strongest electrostatic influence.

The two residues identified here provide additional insight into second coordination sphere effects in LPMOs. In NcAA9C, a well-known second-sphere residue is Gln164, which fine-tunes copper reactivity and influences LPMO functionality.^82^ Although this residue was included in the present calculations, its electrostatic effect on the catalytic core is relatively small compared to Asp76. This reflects the neutral character of the glutamine side chain, whose role is primarily associated with hydrogen-bonding interactions and substrate positioning rather than a strong ionic electrostatic stabilization. The dominant stabilizer is instead Asp76, which lies on the flexible L3 loop rather than at the conserved second-sphere position and has not previously been highlighted in LPMOs.

Glu68 in BaAA10A corresponds to the conserved second-sphere position at which a glutamine or glutamate is structurally conserved in over 96% of AA9, AA10 and AA11 sequences; among AA10s this residue sits at either of two backbone positions, with the side-chain headgroup falling in approximately the same place relative to the copper in both cases.^82^ BaAA10A carries it at the same backbone position as SmAA10A, whose Glu60 was shown to position and constrain H_2_O_2_ and the hydroxyl radical in the peroxygenase reaction.^83^ That the electrostatic perturbation analysis recovers this residue without prior assumption provides an independent check on the approach. What the present simulations add concerns the resting state rather than the reactive complex. In the absence of substrate and cosubstrate, Glu68 hydrogen-bonds the copper-bound equatorial water in 77% of frames, holding it at the equatorial position more often than the metal does, which accounts for the near-complete occupancy and the low exchange rate of that ligand. The equatorial position is therefore constrained by the same carboxylate before and during turnover.

The stabilizing and polarizing effects of Glu and Asp residues at distances of 4–8 Å indicate their relevance for further catalytic LPMO studies, where they need to be explicitly considered. This has not been reported before. Taken together, these observations suggest that the dominant stabilizing effects identified in this analysis arise primarily from the closest charged residue to the copper, whether that residue occupies the conserved second-sphere position, as Glu68 in BaAA10A does, or a different one, as Asp76 in NcAA9C does. Asp76 in particular may represent a target for future investigations into its role in LPMO activity and substrate specificity.

### EPR Analysis of Cu­(II) Species

EPR spectroscopy provides the most direct experimental probe of the oxidized copper site, and both LPMOs studied here have been characterized by it. The g-tensor of a d^9^ center is sensitive to the ligand field in the equatorial plane, while the isotropic copper hyperfine coupling additionally reports on the spin density at the nucleus, so the two observables reveal different aspects of the coordination geometry. Reported experimental values are g_*z*_ = 2.267 and |A_*z*_| = 152 × 10^–4^ cm^–1^ for NcAA9C^37^ and g_*z*_ = 2.250 and |A_*z*_| = 135 × 10^–4^ cm^–1^ for BaAA10A.^38^ The calculated means of EPR parameters for all ten snapshots are collected in Table 3; results for all functionals tested are given in Supplementary Tables S6–S7.

**Table 3:** Calculated EPR Parameters for the QM/MM-Optimized Snapshots, with Deviations from Experiments Given in Parentheses^a^

Enzyme	Structures	n	g_*z*_	|A_*z*_| (10^–4^ cm^–1^)
NcAA9C	All snapshots	10	2.243 ± 0.013	137 ± 37
	A_iso_ **<** 0	8	-	153 ± 16
	Experiment		2.267	152
BaAA10A	All snapshots	10	2.237 ± 0.008	93 ± 28
	A_iso_ **<** 0	2	-	140 ± 10
	Crystal structure	1	2.241	123
	Experiment		2.250	135

a The second row for each enzyme is restricted to those structures whose isotropic coupling retains the physical, negative sign; a positive A_iso_ indicates that the Fermi contact term has collapsed, which occurs when the copper is more than approximately 0.23 Å out of the plane of its donors. No corresponding separating criterion was identified for g_*z*_. The BaAA10A crystal structure (PDB 5IJU) was treated in the same way but is not included in any mean; NcAA9C has no Cu­(II) crystal form, since 4D7U is Cu­(I)-derived.

Previous computational studies of LPMOs and related copper enzymes have reported g_*z*_ values in the range 2.24–2.28 for Cu­(II) active-site models, depending on coordination geometry and the degree of axial distortion.^20,66,84^ The values obtained here, 2.243 and 2.237, fall within that range. Those studies also concluded that reproducing experimental g-tensors requires a realistic treatment of the histidine brace geometry and the inclusion of second-sphere ligands, particularly water molecules.^35,66^ Our perturbation analysis below quantifies that requirement.

Averaged over the ten snapshots, PBE0-DH underestimates g_*z*_ by 0.024 (NcAA9C) and 0.013 (BaAA10A). A systematic underestimation is expected from the functional itself. Benchmark calculations at this level on 18 structurally characterized Cu­(II) complexes report a mean deviation of −0.009.^66^ For BaAA10A the deviation of average g_*z*_ values from the experiment is of that magnitude, whereas for NcAA9C it is roughly twice as large, and this difference is what reduces the calculated separation between the two enzymes to +0.006 ± 0.005, against +0.017 observed experimentally. The g-tensor alone therefore does not discriminate between the two active sites at this level of sampling.

The copper hyperfine coupling is far more informative, and it varies strongly across the structures. Figure 4 shows that the two observables respond to different structural coordinates: g_*z*_ increases with the tetrahedral distortion of the equatorial plane (τ_4_; Pearson correlation coefficient r = +0.67 and +0.69), whereas |A_*z*_| decreases steeply with the displacement of the copper from the plane of its four shortest donors (d_Cu_; r = −0.89 and −0.91). The two descriptors are not independent. Displacing copper from the donor plane raises τ_4_, but τ_4_ responds in addition to twisting of the donor set. They are strongly correlated in NcAA9C (r = 0.91) but only weakly in BaAA10A (0.43); BaAA10A g_*z*_ values correlate with τ_4_ but not with d_Cu_ (+0.08), while |A_*z*_| values correlate with d_Cu_ but not with τ_4_ (−0.10). A relationship between the geometry of the copper site and its EPR parameters has also been reported for the AA10 LPMO from *Photorhabdus luminescens*, where larger values of the geometry index τ were associated with greater $d_{z^{2}}$ mixing into the singly occupied orbital and correspondingly more rhombic parameters.^84^ The origin of the |A_*z*_| dependence is visible in the decomposition of the hyperfine tensor. The spin-dipolar and orbital contributions are almost constant across the snapshots, whereas the Fermi contact term collapses from −232 MHz to +27 MHz, changing sign, as the copper leaves the equatorial plane. Consequently, the isotropic coupling A_iso_, which is necessarily negative for a planar-type Cu­(II) site, changes sign at d_Cu_ ≈ 0.23 Å. Eight of the ten NcAA9C snapshots and two of the ten for BaAA10A retain the planar-type site. In NcAA9C, Tyr166 provides a genuine axial donor, so the site is tetragonally elongated about a well-defined equatorial plane and the copper remains close to it even when one aqua ligand distance increases. BaAA10A has Phe196 in that position, which donates no electron density; both aqua ligands are correspondingly held close and the resulting five-coordinate site distorts toward trigonal bipyramidal, displacing the copper from the plane. For the structures that retain a negative A_iso_, the calculated |A_*z*_| is 153 ± 16 × 10^–4^ cm^–1^ for NcAA9C and 140 ± 10 × 10^–4^ cm^–1^ for BaAA10A, against experimental values of 152 × 10^–4^ cm^–1^ and 135 × 10^–4^ cm^–1^, which give difference between two enzymes close to the experimental values (13 × 10^–4^ cm^–1^ against 17 × 10^–4^ cm^–1^). Averaged over all ten snapshots, the mean values are 137 × 10^–4^ cm^–1^ for NcAA9C and 93 × 10^–4^ cm^–1^ for BaAA10A (Table 3). The residual deviations of the restricted sets, +1 × 10^–4^ cm^–1^ and +5 × 10^–4^ cm^–1^, correspond to 0.6% and 3.4%, well within the mean absolute deviation of 8.1% reported for this functional on the largest hyperfine component across 20 Cu­(II) complexes;^72^ over all ten snapshots the deviations are 10% and 31%. The hyperfine calculation is therefore accurate for structures that describe the observed species, and the deviation of the ten-snapshot means arises from including different copper coordination geometries sampled during MD simulation. For BaAA10A, however, this also points to a limitation of the conformational ensemble itself. Eight of the ten snapshots are displaced from the equatorial plane toward a trigonal-bipyramidal geometry whose electronic structure does not correspond to the rhombic species observed by EPR, indicating that the MD-derived ensemble for this enzyme under-samples the planar-type coordination that predominates under experimental conditions. We note that the BaAA10A subset comprises only two structures, so its standard deviation is indicative rather than statistical; the Cu­(II) crystal structure, treated identically but excluded from all means, provides an independent third point at 123 × 10^–4^ cm^–1.^

**Figure 4:**
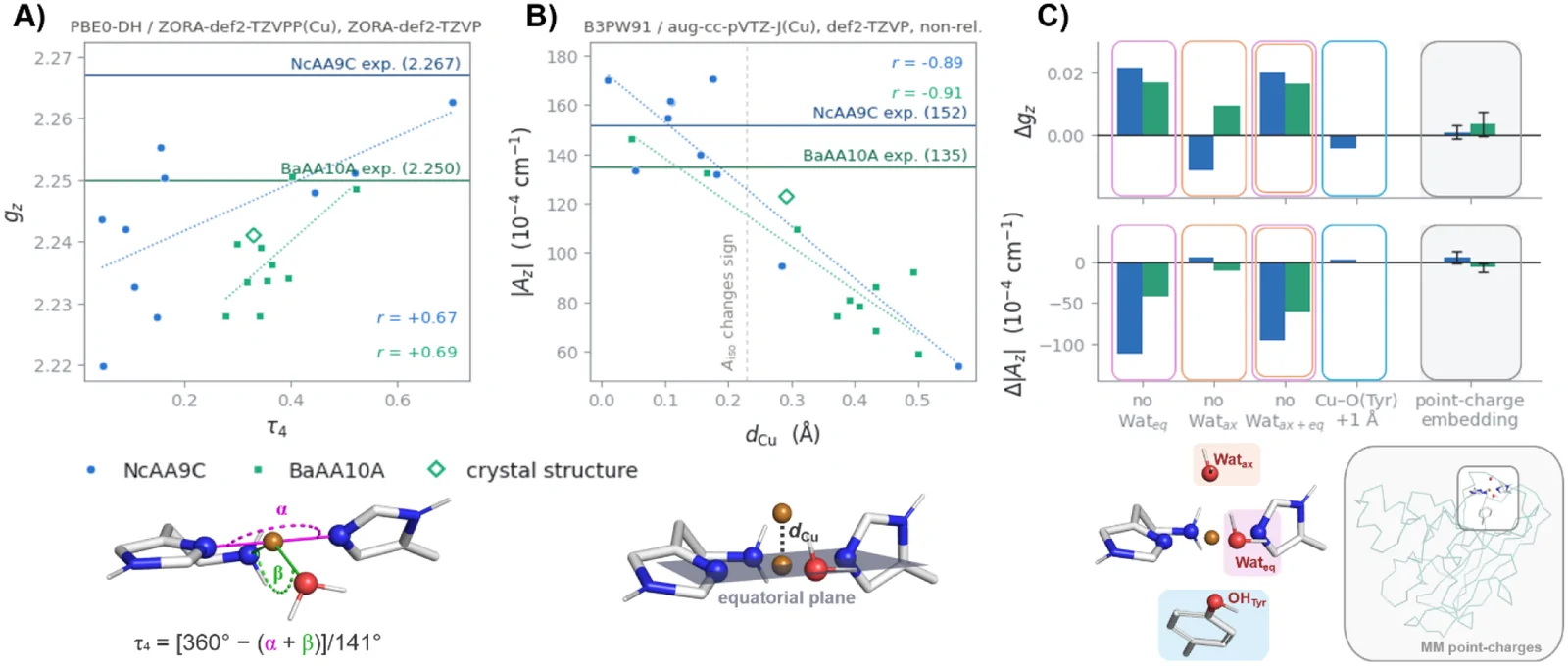
Sensitivity of the calculated Cu­(II) EPR parameters to the active-site geometry. (A) g*_z_* against the tetrahedral distortion parameter τ_4_ and (B) |A*_z_* | against d_Cu_, the displacement of the copper from the equatorial plane, for the ten QM/MM-optimized MD snapshots of each enzyme. NcAA9C, blue circles; BaAA10A, green squares; open green diamond, the BaAA10A Cu­(II) crystal structure (PDB 5IJU), treated identically but excluded from the fits and from all means. Horizontal lines mark the experimental values;^37,38^ dotted lines are least-squares fits over the ten snapshots, with Pearson coefficients annotated. The vertical dashed line marks d_Cu_ = 0.23 Å, where the calculated A_iso_ changes sign. (C) Change in g*_z_* and |A*_z_* | on deleting individual water ligands from, or lengthening Cu–O­(Tyr166) to 3.5 Å (NcAA9C only) in, a single representative snapshot of each enzyme, and on adding the MM point charges, the latter averaged over all ten snapshots and shown with standard deviation. Structures beneath the panels illustrate the two descriptors and the perturbed ligands.

The ^14^N superhyperfine couplings provide a further check. The three coordinating nitrogens are between 10.7 and 15.8 × 10^–4^ cm^–1^ with a mean of 12.5 ± 1.3 × 10^–4^ cm^–1^, which is an excellent agreement with the experimental splitting constant of ≈13 × 10^–4^ cm^–1^ reported for NcAA9C.^37^ Individual nitrogen nuclei could not be resolved and further double resonance experiments are needed to allow a definite assignment. For BaAA10A, experimental ^14^N superhyperfine coupling constants are not resolved and our calculated mean value is 12.7 ± 0.5 × 10^–4^ cm^–1^.

To identify which ligand dominates the EPR observables, single-point calculations were repeated on clusters in which one ligand at a time was altered (Supplementary Table S8, Figure 4C). Removing the equatorial water shifts g_*z*_ by +0.021, reduces |A_*z*_| from 134 to 21 × 10^–4^ cm^–1^ and reduces the imidazole ^14^N couplings by a third. Removing the axial water has only a minor effect on the hyperfine coupling constant, raising |A_*z*_| by 7 × 10^–4^ cm^–1^, although it shifts g_*z*_ by −0.011, about half the equatorial water effect. The same ordering is found for BaAA10A. Increasing Cu–O­(Tyr166) distance in NcAA9C from 2.54 to 3.50 Å changes g_*z*_ by −0.004, a third of the standard deviation across the ten snapshots, and |A_*z*_| by 4 × 10^–4^ cm^–1^. The axial tyrosine in NcAA9C is therefore spectroscopically almost undetectable, and the difference in EPR parameters between the two families does not originate from it. The equatorial water therefore proves decisive at every stage of this study: its position is not fully accurately determined by the classical force fields, but it dominates both EPR observables.

Embedding the QM cluster in the point-charge field of the complete protein changes g_*z*_ by 0.001–0.004 and |A_*z*_| by 6 × 10^–4^ cm^–1^ (Supplementary Tables S6–S7, Figure 4C), in each case smaller than the spread across ten snapshots. The QM region used here therefore already contains the electrostatics that determine the copper g- and A-tensors. The one observable that does respond is the ^14^N coupling of the N-terminal amine, the donor least shielded by the QM region, which decreases from 15.8 × 10^–4^ cm^–1^ to 14.8 × 10^–4^ cm^–1^ in NcAA9C and from 14.9 × 10^–4^ cm^–1^ to 13.2 × 10^–4^ cm^–1^ in BaAA10A, while the two imidazole nitrogens change by 0.6 × 10^–4^ cm^–1^ at most.

## Conclusions

This study characterizes how copper oxidation state, second-sphere coordination environment and local protein context jointly modulate the geometry, stability and dynamics of the histidine brace in LPMO enzymes, combining MD simulations with QM/MM optimization and the calculation of EPR parameters. In our MD simulations, the brace itself is described by bonded parameters and remains close to its parametrized geometry throughout, so the structural response to the change in oxidation state is carried almost entirely by the ligands treated without bonded terms. Subsequent QM/MM optimizations confirm the trends identified in the MD simulations. We also provide validated MD parameters for the histidine brace in both the AA9 and AA10 families, in the oxidized and reduced states. Direct validation on further family members would strengthen the case for general applicability of those parameters.

Our simulations confirm that structural changes upon oxidation are dominated by copper–water interactions rather than by rearrangements of the histidine brace itself. Cu­(II) displays tighter water coordination, with shorter Cu–O distances and higher occupancy, whereas Cu­(I) exhibits weaker and more labile hydration. In BaAA10A the equatorial water is additionally anchored by a second-sphere glutamate, hydrogen-bonded in 77% of frames, which has no counterpart in NcAA9C and accounts for near-complete water occupancy and an exchange rate more than an order of magnitude lower than in either the reduced state or the AA9 enzyme. Beyond structural characterization, our electrostatic perturbation analysis identifies Asp76 in NcAA9C and Glu68 in BaAA10A as the principal residues stabilizing the histidine brace via water-mediated interactions. The stronger stabilizing effect observed in the Cu­(II) state underscores the importance of second-sphere electrostatics in supporting the higher oxidation state and suggests a conserved functional role for acidic residues in modulating copper coordination across LPMO families. Both residues should therefore be included in the QM region of mechanistic studies. Glu68 is already recognized in BaAA10A as a second-sphere residue that stabilizes the cosubstrate and here it anchors the equatorial water. The role of Asp76 in NcAA9C, however, should be further investigated.

EPR g-tensors, copper hyperfine couplings and ^14^N superhyperfine couplings computed from the selected snapshots reveal that the two copper observables respond to different geometric coordinates (g_*z*_ to the tetrahedral distortion of the equatorial plane and |A_*z*_| to the displacement of the copper from it), providing a structure–property relationship rather than a single point of comparison with experiment. The axial tyrosine of NcAA9C, despite lying within coordination distance, is spectroscopically almost undetectable, even when displaced by 1 Å. The difference in EPR parameters between the AA9 and AA10 families therefore probably does not originate from the ligand at the axial position, but from the position of the equatorial aqua ligand and the distortion from planar geometry.

## Supplementary Material



## Data Availability

The data underlying this study are openly available in Zenodo repository at 10.5281/zenodo.22640262.

## Notes

MCPB-derived partial charges and force field parameters for the histidine brace; copper coordination geometry, water occupancy, and exchange statistics from MD simulations with statistical analysis; MD trajectory clustering and QM/MM optimization effects; multiple sequence alignments of AA9 and AA10 LPMOs highlighting conserved second-sphere residues (PDF)

## #

Department of Chemistry, University of Delhi (North Campus), Delhi 110007, India

## Abbreviations

LPMO: lytic polysaccharide monooxygenases
AA: auxiliary activity
MD: molecular dynamics
QM/MM: quantum mechanics/molecular mechanics
DFT: density functional theory
SCF: self-consistent field
EPR: electron paramagnetic resonance
RMSF: root-mean-square fluctuation
RMSD: root-mean-square deviation.
