# Supplementary material for: Multiscale Modeling of Lytic Polysaccharide Monooxygenases in Oxidized and Reduced Redox States
Source: J Phys Chem B. 2026 Oct 15;130(43):11369–82. doi: 10.1021/acs.jpcb.6c04364 (PMC13726380; doi:10.1021/acs.jpcb.6c04364)
Supplement: Supplementary file 1 [file jp6c04364_si_001.pdf]

Supporting Information

Multiscale Modelling of Lytic Polysaccharide Monooxygenases in Oxidized and Reduced Redox States

Katarzyna Szleper-Shendy[a,b], Andrés M. Escorcia[b], Ravi Kumar[b], Artur Góra[a], Matthias Stein[b]

[a] Tunneling Group, Biotechnology Centre, Silesian University of Technology, Gliwice, Poland

[b] Molecular Simulations and Design Group, Max Planck Institute for Dynamics of Complex Technical Systems, Magdeburg, Germany

Supplementary Table S1. MCPB-derived partial charges for the histidine brace in Cu(I) and Cu(II) states. HIX corresponds to the N-terminal histidine coordinating copper through the histidine brace, and HIY denotes the second coordinating histidine.

residue	atom name	atom type	Cu(I) charges	Cu(II) charges
HIX	N	Y1	0,053	-0,358
	H1	H	0,102	0,228
	H2	H	0,102	0,228
	CA	CX	-0,294	-0,253
	HA	HP	0,095	0,145
	CB	CT	-0,272	-0,337
	HB2	HC	0,149	0,178
	HB3	HC	0,149	0,178
	CG	CC	0,246	0,239
	ND1	Y2	-0,100	-0,149
	CE1	CR	-0,134	-0,100
	HE1	H5	0,188	0,215
	NE2	NA	-0,057	-0,087
	HE2	H	0,318	0,353
	CD2	CW	-0,338	-0,232
	HD2	H4	0,221	0,232
	C	C	0,740	0,869
	O	O	-0,566	-0,527
HIY	N	N	-0,416	-0,416
	H	H	0,294	0,363
	CA	XC	0,019	0,019
	HA	H1	0,091	0,173
	CB	CT	0,180	0,745
	HB2	HC	0,017	-0,096

	HB3	HC	0,017	-0,096
	CG	CC	-0,173	-0,571
	ND1	NA	-0,051	0,186
	HD1	H	0,292	0,208
	CE1	CR	-0,058	-0,024
	HE1	H5	0,165	0,169
	NE2	Y3	-0,060	-0,136
	CD2	CV	-0,180	-0,044
	HD2	H4	0,190	0,171
	C	C	0,597	0,597
	O	O	-0,568	-0,568
CU	CU	M1	0,041	0,496
total charge			1,000	2,000

Supplementary Table S2. MCPB-derived force field parameters for the histidine brace in Cu(I) and Cu(II) states. Only parameters that differ between the two oxidation states are reported; all remaining force field terms are identical in both models and are provided in the repository.

BOND LENGTH PARAMETERS				
atom types	harmonic force constant [kcal/mol/Å^2]	equilibrium bond length [Å]	harmonic force constant [kcal/mol/Å^2]	equilibrium bond length [Å]
	Cu(I)		Cu(II)	
Y1-M1	7.80	2.26	49.40	2.03
Y2-M1	83.30	1.95	130.10	1.91
Y3-M1	89.20	1.94	128.20	1.90
**BOND ANGLE PARAMETERS**				
atom types	harmonic force constants for the angle [kcal/mol/rad^2]	equilibrium bond angle [˚]	harmonic force constants for the angle [kcal/mol/rad^2]	equilibrium bond angle [˚]
	Cu(I)		Cu(II)	
CC-Y2-M1	76.70	124.02	96.24	125.21
CR-Y3-M1	67.29	127.38	74.82	126.72
M1-Y1-CX	24.06	114.66	78.86	116.38
M1-Y1-H	29.51	108.26	41.76	106.67
M1-Y2-CR	74.86	128.49	90.69	127.07
M1-Y3-CV	68.63	126.20	71.97	126.35
Y1-M1-Y2 (θ_1)	19.45	95.40	66.01	97.06
Y1-M1-Y3 (θ_2)	20.00	109.37	42.82	103.50
Y2-M1-Y3 (θ_3)	29.15	155.22	44.00	159.42

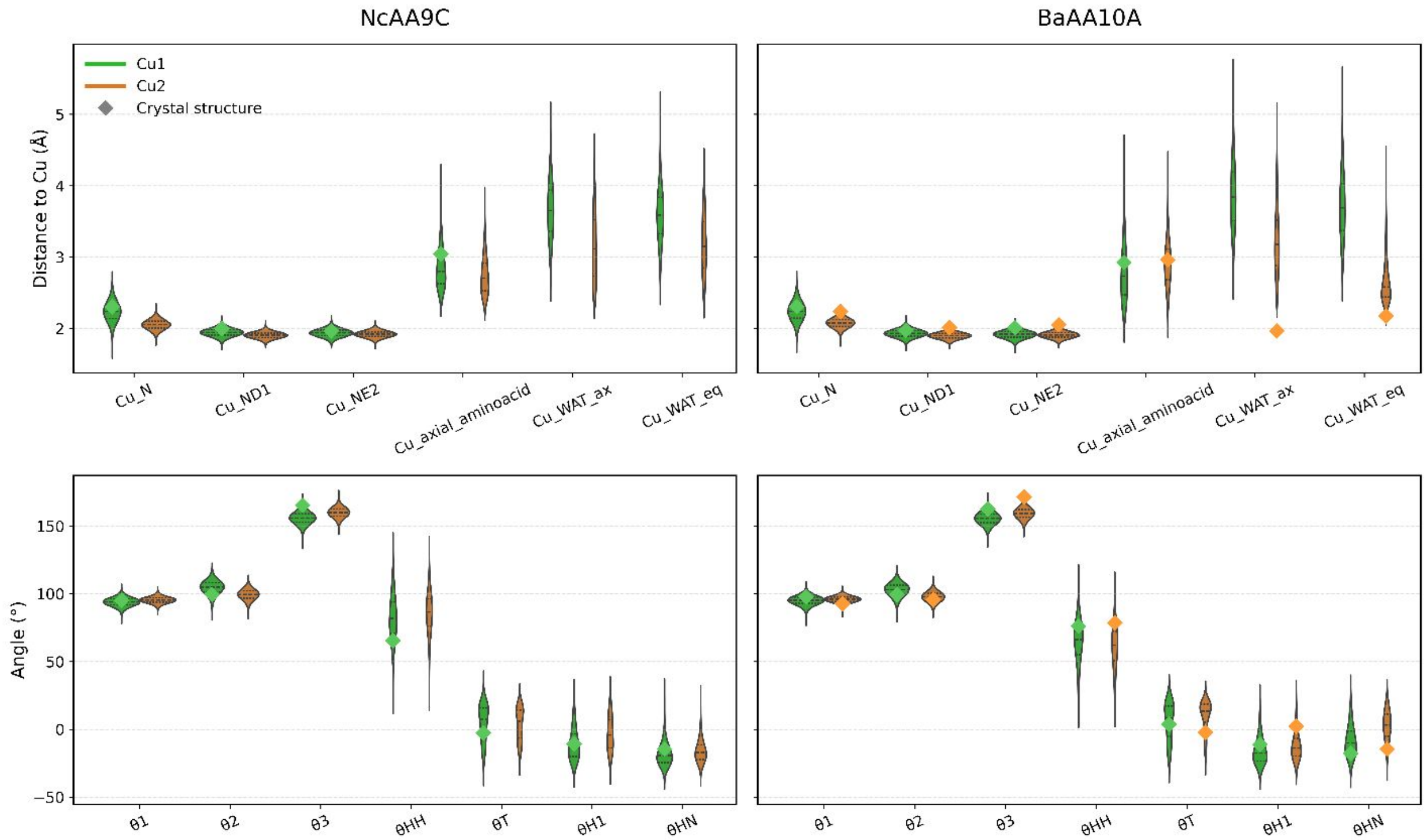

Supplementary Figure S1. Copper coordination geometry parameters for NcAA9C (left column) and BaAA10A (right column). The top row shows Cu-ligand distances, and the bottom row shows coordination angles (θ) from molecular dynamics simulations. Results for systems with Cu(I) and Cu(II) are shown in green and orange, respectively. Diamond symbols indicate values calculated from the corresponding crystal structures.

Supplementary Table S3. Statistical comparison of histidine-brace geometries from MD simulations. Differences in Cu-ligand distances and coordination angles between Cu(I) and Cu(II) within each protein, as well as between NcAA9C and BaAA10A for each copper state, were assessed using the nonparametric, two-sided Mann-Whitney U test. Resulting p-values were corrected for multiple testing using the Benjamini-Hochberg false discovery rate (FDR) procedure. Effect sizes were estimated from median differences, with thresholds of 0.1 Å for distances and 5° for angles to identify practically meaningful differences.

Protein	Comparison	Parameter	p-value	p-adjusted	Median difference	Effect size
NcAA9C	Cu1 vs Cu2	Cu_N	<1e-10	<1e-10	-0,18	meaningful
		Cu_ND1	$<1e^{-10}$	<1e-10	-0,04	not meaningful
		Cu_NE2	<1e-10	<1e-10	-0,02	not meaningful
		Cu_axial_aminoacid	<1e-10	<1e-10	-0,09	not meaningful
		Cu_WAT_ax	<1e-10	<1e-10	-0,54	meaningful
		Cu_WAT_eq	<1e-10	<1e-10	-0,44	meaningful
		θ1	<1e-10	<1e-10	1,43	not meaningful
		θ2	<1e-10	<1e-10	-5,37	meaningful
		θ3	<1e-10	<1e-10	4,07	not meaningful
		θHH	<1e-10	<1e-10	4,82	not meaningful
		θT	<1e-10	<1e-10	-1,39	not

						meaningful
		θH1	<1e-10	<1e-10	8,74	meaningful
		θHN	<1e-10	<1e-10	2,31	not meaningful
BaAA10A		Cu_N	<1e-10	<1e-10	-0,16	meaningful
		Cu_ND1	<1e-10	<1e-10	-0,03	not meaningful
		Cu_NE2	<1e-10	<1e-10	-0,01	not meaningful
		Cu_axial_aminoacid	<1e-10	<1e-10	0,16	meaningful
		Cu_WAT_ax	<1e-10	<1e-10	-0,67	meaningful
		Cu_WAT_eq	<1e-10	<1e-10	-1,11	meaningful
		θ1	<1e-10	<1e-10	0,73	not meaningful
		θ2	<1e-10	<1e-10	-5,42	meaningful
		θ3	<1e-10	<1e-10	3,68	not meaningful
		θHH	<1e-10	<1e-10	-4,23	not meaningful
		θT	<1e-10	<1e-10	5,18	meaningful
		θH1	<1e-10	<1e-10	3,74	not meaningful
		θHN	<1e-10	<1e-10	13,36	meaningful
NcAA9C vs BaAA10A	Cu1	Cu_N	4,07E-02	4,07E-02	0	not meaningful
		Cu_ND1	<1e-10	<1e-10	-0,01	not meaningful
		Cu_NE2	<1e-10	<1e-10	-0,02	not meaningful

		Cu_axial_aminoacid	<1e-10	<1e-10	-0,07	not meaningful
		Cu_WAT_ax	<1e-10	<1e-10	0,19	meaningful
		Cu_WAT_eq	<1e-10	<1e-10	0,1	meaningful
		θ1	<1e-10	<1e-10	1,24	not meaningful
		θ2	<1e-10	<1e-10	-1,83	not meaningful
		θ3	7,98E-10	8,14E-10	-0,24	not meaningful
		θHH	<1e-10	<1e-10	-15,45	meaningful
		θT	<1e-10	<1e-10	1,02	not meaningful
		θH1	<1e-10	<1e-10	-4,57	not meaningful
		θHN	<1e-10	<1e-10	9,31	meaningful
	Cu2	Cu_N	<1e-10	<1e-10	0,02	not meaningful
		Cu_ND1	<1e-10	<1e-10	-0,01	not meaningful
		Cu_NE2	<1e-10	<1e-10	-0,01	not meaningful
		Cu_axial_aminoacid	<1e-10	<1e-10	0,19	meaningful
		Cu_WAT_ax	<1e-10	<1e-10	0,06	not meaningful
		Cu_WAT_eq	<1e-10	<1e-10	-0,57	meaningful
		θ1	<1e-10	<1e-10	0,54	not meaningful
		θ2	<1e-10	<1e-10	-1,88	not meaningful

		θ3	<1e-10	<1e-10	-0,63	not meaningful
		θHH	<1e-10	<1e-10	-24,49	meaningful
		θT	<1e-10	<1e-10	7,59	meaningful
		θH1	<1e-10	<1e-10	-9,57	meaningful
		θHN	<1e-10	<1e-10	20,37	meaningful

Supplementary Table S4. Fraction of MD trajectory frames in which a water molecule occupies the axial or equatorial position within 3.5 Å of the copper center in NcAA9C and BaAA10A in the Cu(I) and Cu(II) states. Percentages are calculated over the full production trajectories (total of 30 000 frames) and reflect the relative occupancy of axial (WAT_ax) and equatorial (WAT_eq) water ligands within the first coordination shell of copper.

System	WAT_ax ≤3.5 Å	WAT_eq ≤3.5 Å
NcAA9C Cu(I)	36.2%	41.2%
NcAA9C Cu(II)	73.7%	75.3%
BaAA10A Cu(I)	24.3%	34.6%
BaAA10A Cu(II)	74.1%	97.4%

Supplementary Table S5. Number of exchange events for the nearest axial and equatorial water molecules during the MD simulations of NcAA9C and BaAA10A in the Cu(I) and Cu(II) states. Exchange events are defined as changes of the closest water molecule residues between consecutive trajectory frames, without applying any distance cutoff, providing a measure of water ligand mobility and coordination stability at the copper active site.

System	Exchange count	
	WAT_ax	WAT_eq
NcAA9C Cu(I)	21108	18124
NcAA9C Cu(II)	15436	15977
BaAA10A Cu(I)	19491	15146
BaAA10A Cu(II)	10420	1225

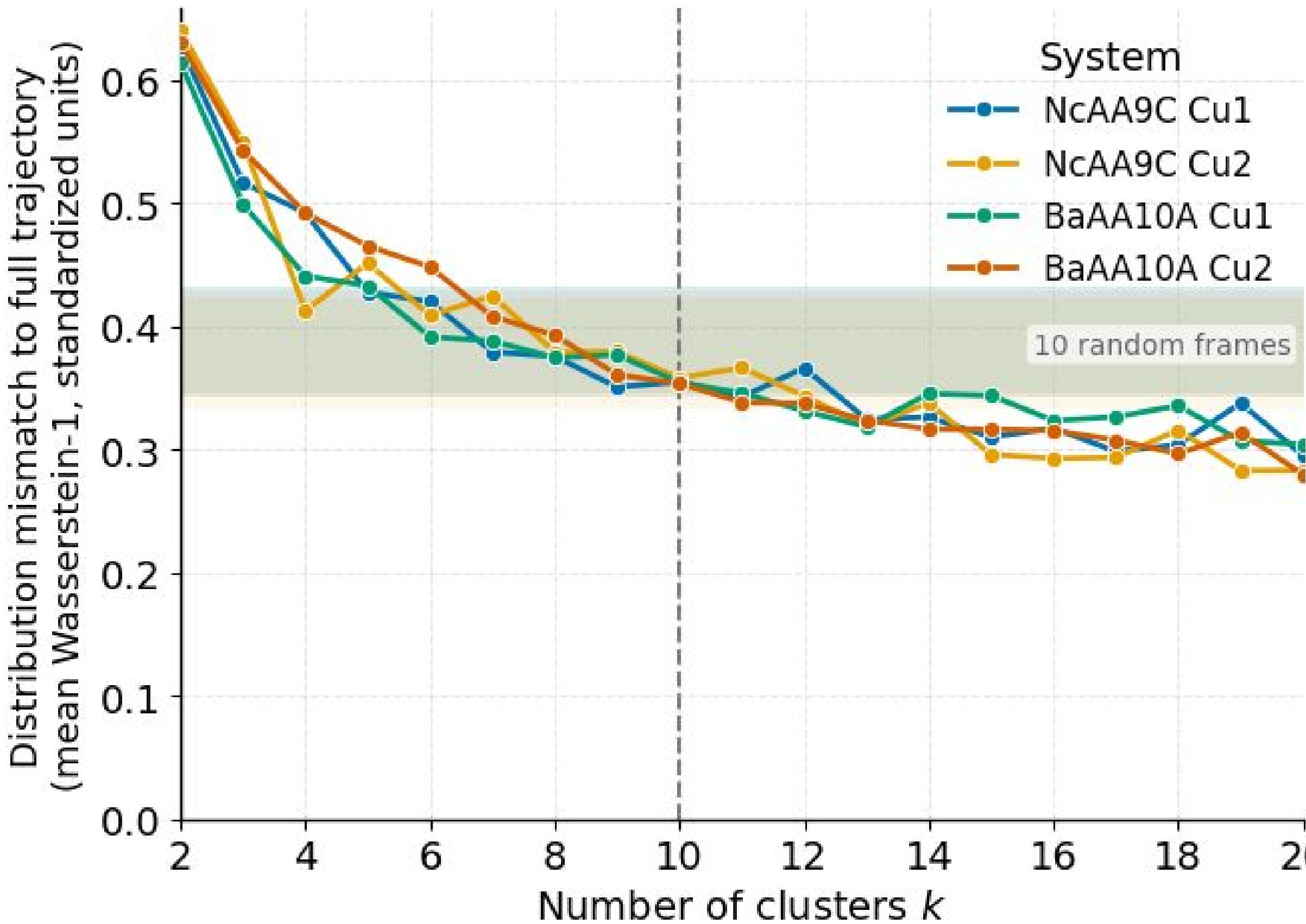

Supplementary Figure S2. Convergence of the representative-structure set with the number of clusters k. Each concatenated production trajectory (3 × 100 ns, 30 000 frames) was clustered by k-means on 13 standardized active-site descriptors (see Methods). At each k, the population-weighted set of cluster medoids was compared with the full trajectory using the 1-Wasserstein distance between descriptor distributions, averaged over all 13 (lower values indicate closer agreement; standardized units). Dashed line, k = 10, used throughout this work; shaded bands, mismatch obtained from ten randomly selected frames (mean ± one standard deviation over 300 repetitions). The mismatch falls steeply to k ≈ 8–10 and flattens thereafter, the gain per additional cluster dropping from about 0.033 below k = 10 to below 0.01 above it, comparable to the seed-to-seed variability of the clustering itself. All four systems behave similarly.

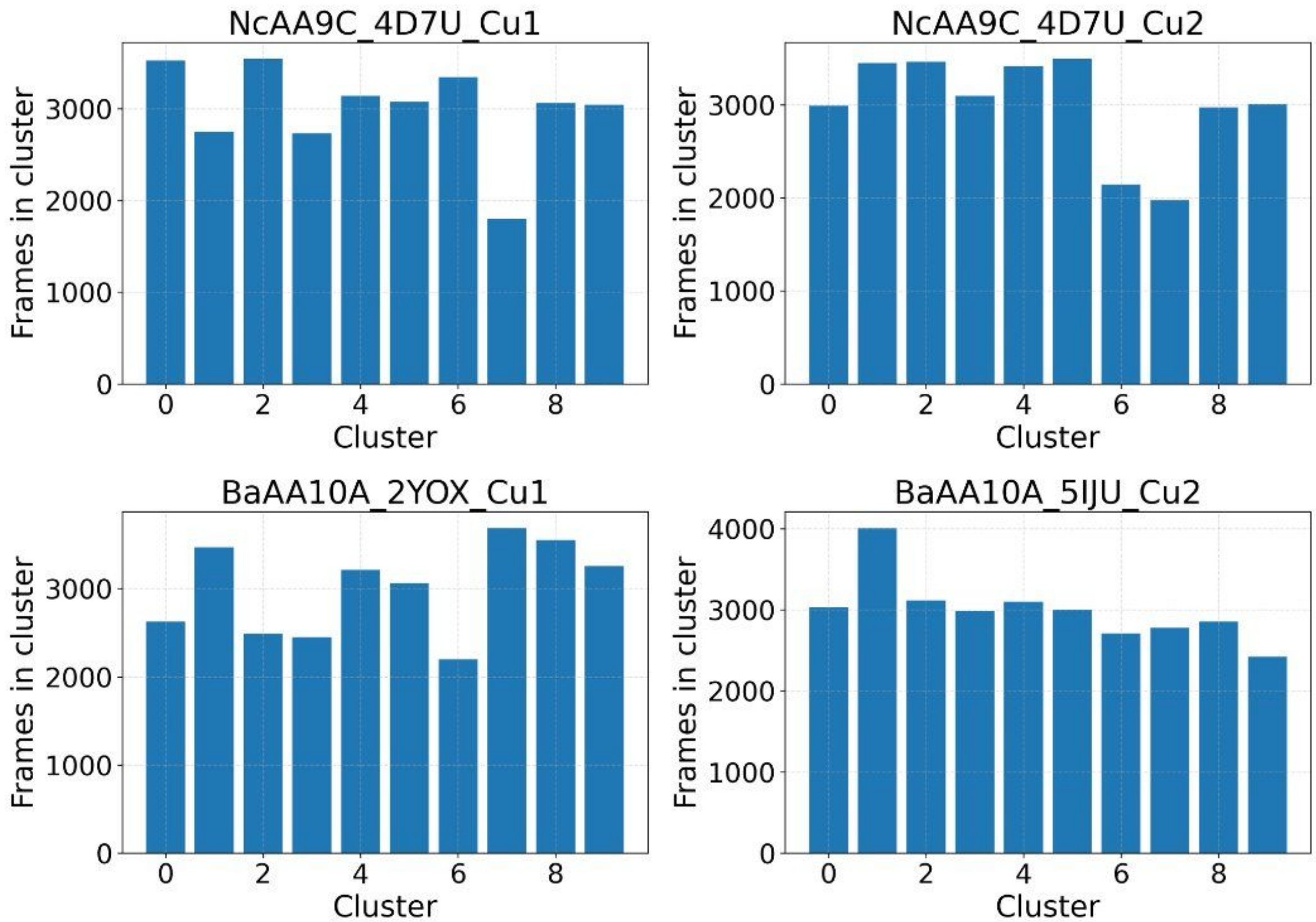

Supplementary Figure S3. Populations of clusters.

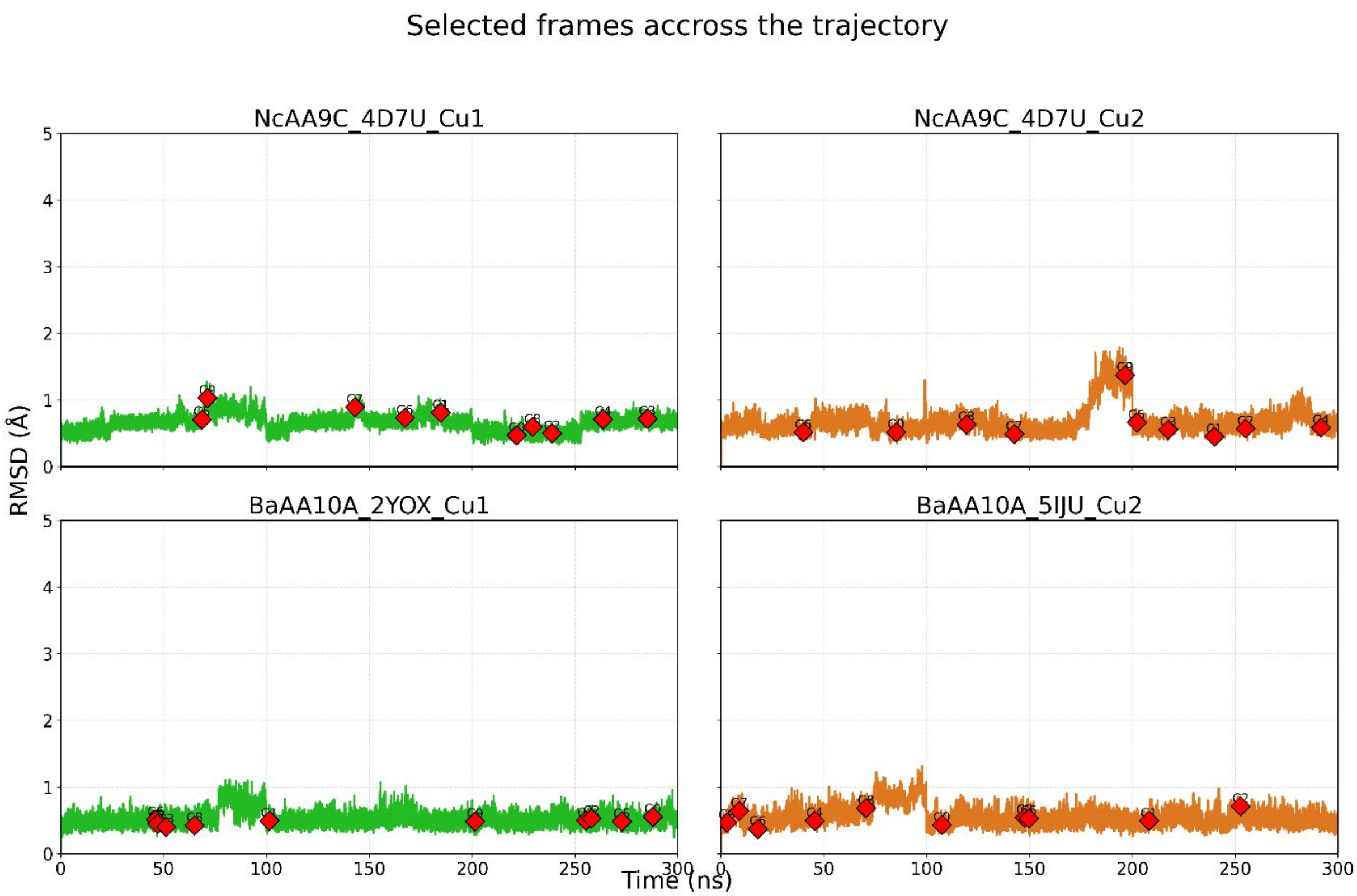

Supplementary Figure S4. Location of selected frames as cluster representatives across the MD trajectory.

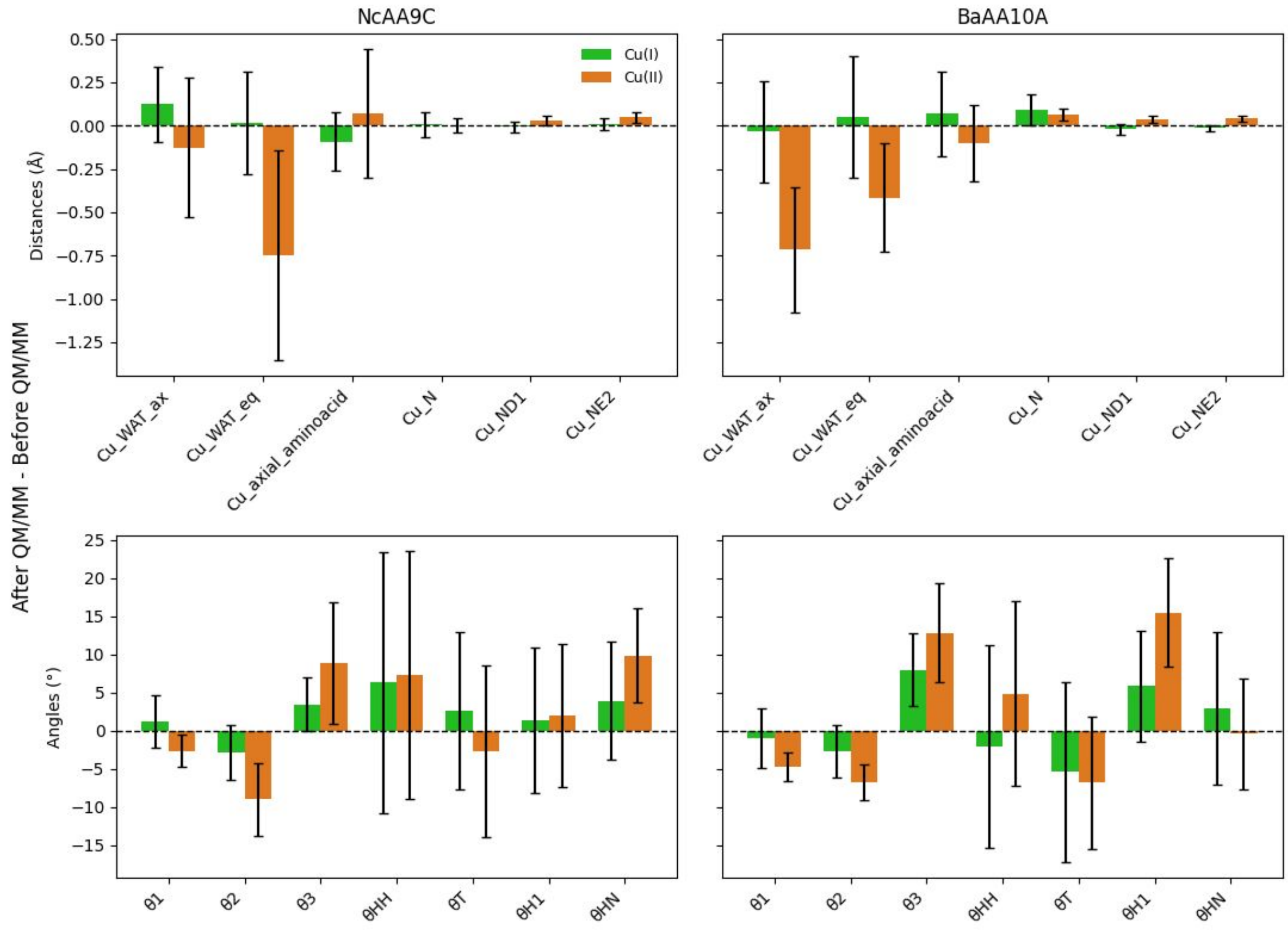

Supplementary Figure S5. Differences in copper coordination geometry before and after QM/MM optimization. Bar plots show the average change (± standard deviation) for selected frames from MD simulations for selected distances (top row) and angles (bottom row) in the histidine brace for Cu(I) and Cu(II) in NcAA9C (left column) and BaAA10A (right column). Green bars correspond to Cu(I) and orange bars to Cu(II). A horizontal dashed line at 0 indicates no change. Positive values represent an increase and negative values a decrease after QM/MM optimization. The bars therefore measure the displacement of each coordinate from the nearest local minimum of the QM/MM potential energy surface: small for the copper-nitrogen distances, which are constrained by bonded parameters, and substantially larger for the water and axial ligands, which are described by non-bonded terms alone.

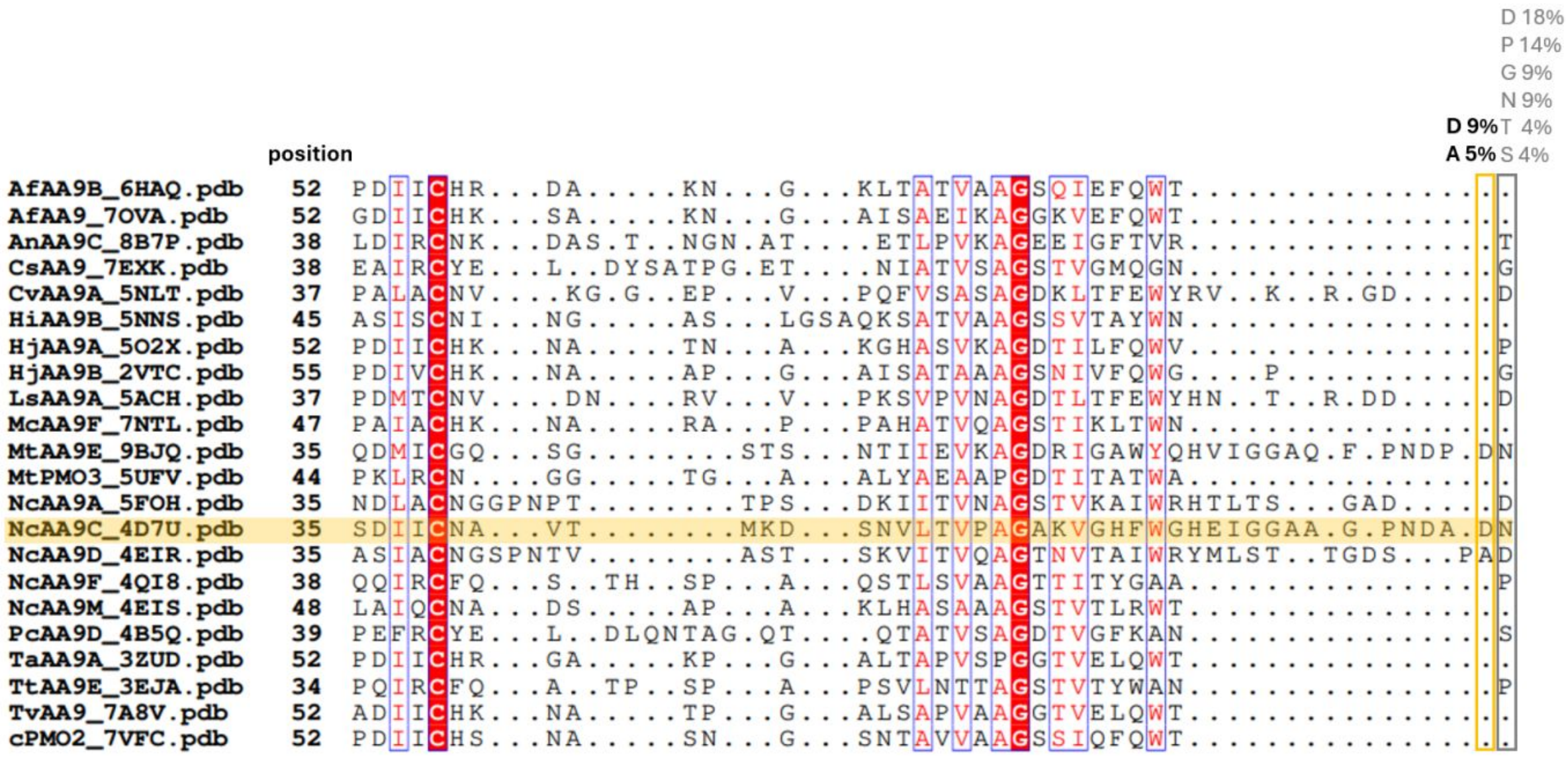

Supplementary Figure S6. Multiple sequence alignment of 22 structurally characterized AA9 LPMOs, focused on the region containing Asp76 in NcAA9C. The alignment was generated using the mTM-align tool, which performs structural alignment, and visualized with ESPript 3.2. The NcAA9C sequence is highlighted in yellow, with the Asp76 residue position also marked in yellow frame. The percentage identity above the alignment indicates the degree of conservation at this position.

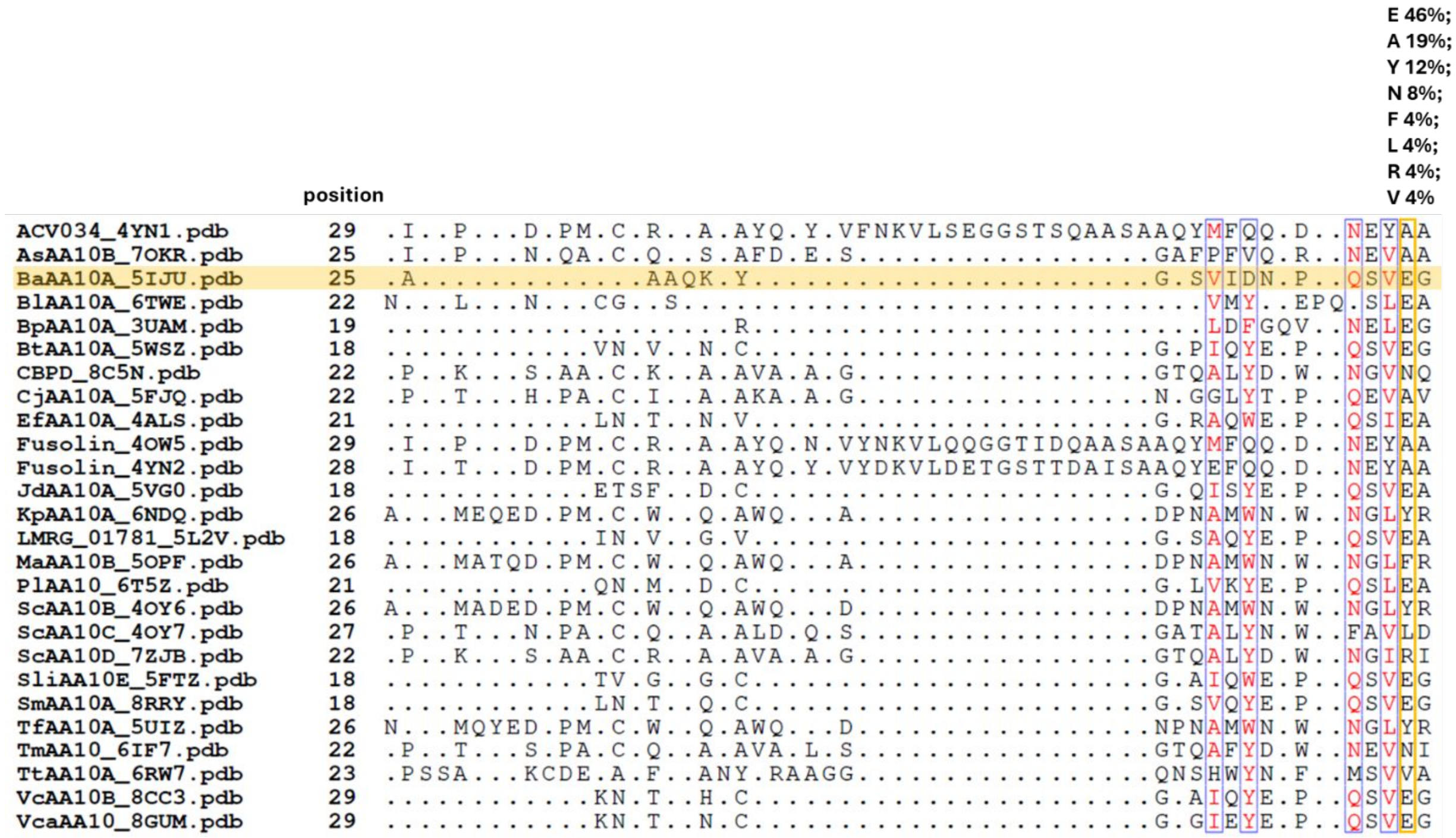

Supplementary Figure S7. Multiple sequence alignment of 26 structurally characterized AA10 LPMOs, focused on the region containing Glu68 in BaAA10A. The alignment was generated using the mTM-align tool, which performs structural alignment, and visualized with ESPript 3.2. The BaAA10A sequence is highlighted in yellow, with the Glu68 residue position also marked in yellow frame. The percentage identity above the alignment indicates the degree of conservation at this position.

Supplementary Table S6. EPR parameters of LPMO NcAA9C. Mean ± standard deviation over the ten QM/MM-optimized snapshots. Nitrogen hyperfine couplings in 10^{-4} cm^{-1}.

Level of theory	g_z	$\lvert A_z \rvert$	$A_{\perp N}$	$A_{\perp ND1}$	$A_{\perp NE2}$	$A_{\perp \text{mean of three}}$
B3PW91/J, non-rel.	2.175 ± 0.007	137 ± 37	15.8 ± 1.0	11.2 ± 1.6	10.7 ± 2.0	12.5 ± 1.3
B3PW91/J, non-rel. + point charges	2.178 ± 0.008	143 ± 40	14.8 ± 1.3	11.8 ± 1.7	10.6 ± 2.1	12.4 ± 1.3
PBE0-DH	2.243 ± 0.013	158 ± 43	14.2 ± 1.0	10.3 ± 1.6	9.7 ± 2.0	11.4 ± 1.3
PBE0-DH + point charges	2.244 ± 0.014	164 ± 46	13.4 ± 1.2	10.7 ± 1.6	9.7 ± 2.1	11.3 ± 1.4
experiment	2.267	152	not resolved	not resolved	not resolved	≈13.0

The B3PW91/J calculations were performed without a scalar relativistic Hamiltonian, as required by the protocol benchmarked for hyperfine couplings; the g-values from these calculations are listed for completeness but are not meaningful, and g_z is taken throughout from the PBE0-DH/ZORA calculations.

Borisova et al. [37] report a single splitting constant, $A \approx 13 \times 10^{-4}$ cm^{-1}, consistent with coupling to nearby nitrogen nuclei; the individual histidine-brace nitrogens are not resolved experimentally.

Supplementary Table S7. EPR parameters of BaAA10A. Mean ± standard deviation over the ten QM/MM-optimized snapshots. Nitrogen hyperfine couplings in 10^{-4} cm^{-1}.

Level of theory	g_z	$\lvert A_z \rvert$	$A_{\perp\,N}$	$A_{\perp\,ND1}$	$A_{\perp\,NE2}$	$A_{\perp\,\text{mean of three}}$
B3PW91/J, non-rel.	2.171 ± 0.005	93 ± 28	14.9 ± 1.1	11.6 ± 0.7	11.5 ± 0.8	12.7 ± 0.5
B3PW91/J, non-rel. + point charges	2.177 ± 0.004	87 ± 32	13.2 ± 1.1	12.1 ± 0.6	11.5 ± 0.7	12.3 ± 0.5
PBE0-DH	2.237 ± 0.008	110 ± 32	13.1 ± 1.0	10.5 ± 0.6	10.3 ± 0.7	11.3 ± 0.5
PBE0-DH + point charges	2.241 ± 0.007	105 ± 35	11.9 ± 1.1	10.7 ± 0.4	10.3 ± 0.6	10.9 ± 0.5
experiment	2.250	135	n.a.	n.a.	n.a.	n.a.

The B3PW91/J calculations were performed without a scalar relativistic Hamiltonian, as required by the protocol benchmarked for hyperfine couplings; the g-values from these calculations are listed for completeness but are not meaningful, and g_z is taken throughout from the PBE0-DH/ZORA calculations.

Supplementary Table S8. Change in the calculated EPR parameters on perturbing one ligand of a representative snapshot of each enzyme, relative to the unperturbed structure. Δg_z is quoted at PBE0-DH and the hyperfine changes at B3PW91/aug-cc-pVTZ-J, each being the level at which that observable is reported. Hyperfine couplings are reported in 10^{-4} cm^{-1}.

**Structure**	**Perturbation**	**PBE0-DH**	**B3PW91/aug-cc-pVTZ-J**			
		Δg_z	ΔA_z	$\Delta A_{\perp N}$	$\Delta A_{\perp ND1}$	$\Delta A_{\perp NE2}$
**NcAA9C snapshot 25502**	unperturbed	2.244	134	15.3	11.9	11.5
	equatorial water removed	2.265	21	16.2	8.0	8.2
	axial water removed	2.233	140	17.0	11.7	11.1
	both waters removed	2.263	38	19.0	6.7	6.8
	Cu–O(Tyr166) 3.0 Å	2.242	137	16.1	11.6	11.2
	Cu–O(Tyr166) 3.5 Å	2.240	137	16.4	11.6	11.2
**BaAA10A snapshot 4556**	unperturbed	2.234	110	14.7	12.0	12.4
	equatorial water removed	2.250	55	18.3	8.7	8.8
	axial water removed	2.238	109	16.3	10.0	11.0
	both waters removed	2.247	47	18.9	6.1	6.7
